# Recent Progress in the Application of Hydroxyapatite for the Adsorption of Heavy Metals from Water Matrices

**DOI:** 10.3390/ma14226898

**Published:** 2021-11-15

**Authors:** Roxana Ioana Brazdis, Irina Fierascu, Sorin Marius Avramescu, Radu Claudiu Fierascu

**Affiliations:** 1 National Institute for Research & Development in Chemistry and Petrochemistry—ICECHIM, 060021 Bucharest, Romania; roxana.brazdis@icechim.ro; 2 Department of Science and Engineering of Oxide Materials and Nanomaterials, University “Politehnica” of Bucharest, 011061 Bucharest, Romania; 3 Faculty of Horticulture, University of Agronomic Sciences and Veterinary Medicine of Bucharest, 011464 Bucharest, Romania; 4 Research Center for Environmental Protection and Waste Management, University of Bucharest, 91-95 Splaiul Independentei, 050095 Bucharest, Romania; sorin_avramescu@yahoo.com; 5 Department of Organic Chemistry, Biochemistry and Catalysis, Faculty of Chemistry, University of Bucharest, 90-92 Soseaua Panduri, 050663 Bucharest, Romania

**Keywords:** water depollution, heavy metals, adsorption materials, natural and synthetic hydroxyapatite

## Abstract

Wastewater treatment remains a critical issue globally, despite various technological advancements and breakthroughs. The study of different materials and technologies gained new valences in the last years, in order to obtain cheap and efficient processes, to obtain a cleaner environment for future generations. In this context, the present review paper presents the new achievements in the materials domain with highlights on apatitic materials used for decontamination of water loaded with heavy metals. The main goal of this review is to present the adsorptive removal of heavy metals using hydroxyapatite-based adsorbents, offering a general overview regarding the recent progress in this particular area. Developing the current review, an attempt has been made to give appropriate recognition to the most recent data regarding the synthesis methods and targeted pollutants, including important information regarding the synthesis methods and precursors, morphological characteristics of the adsorbent materials and effectiveness of processes.

## 1. Introduction

To treat and clean contaminated water is a very difficult process, water pollution representing a critical issue of our society. Industrial and agriculture sectors generate huge quantities of chemical compounds that can cause serious problems of environmental damage, heavy metals pollution being one of it, being more persistent than organic contaminants such as pesticides or petroleum byproducts. Heavy metals can produce serious human health problems (including hyperkeratosis, cancers, diabetes, anemia, disorders of the immune nervous and reproductive systems, etc. [1]).

Today, the challenges in finding new materials and technologies for water depollution are directed towards a balance of operating costs (including easy scale-up of the decontamination procedures and materials synthesis) and contaminant removal efficiency. Remediation techniques, such as adsorption, membrane technology, ion exchange, coagulation or electrochemical treatment, are the most versatile applications, which can be comprehensively upgraded for the remediation behavior of heavy metals contaminated environment. Among the depollution strategies, adsorption is well-recognized as a viable and easy-to-apply at industrial level method for the removal of both organic and inorganic pollutants [2,3]. The research into adsorption materials gained new valences from the study of minerals, biopolymers, microalgal and fungal biomass to waste materials/byproducts or nanotechnology products, in order to obtain heavy metals removal efficiency in acidic, neutral or alkaline conditions [4,5,6,7]. Moreover, it is of great interest to use different innovative materials with good biocompatibility, enhanced possibility of biodegradability and bioreactivity [8].

What is important in the environmental practices of depollution is that the tandem “material–technology” has to offer not only economic benefits, but it has to contribute in achieving a sustainable development by being an active part in the global waste management process. These used materials must be removed from the systems in order to not be considered a threat to plants, animals and even humans because of their bioaccumulation, after their role is fully achieved.

**Scheme 1:**
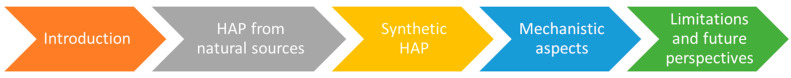
Flowchart describing the structure of the present review.

In this context, this review reports the recent applications of hydroxyapatite and hydroxyapatite-based materials in the area of heavy metals removal. Due to their chemical characteristics (enhanced thermal and chemical stability, acid-base properties, low solubility, adsorption and ion-exchange ability), these materials can be an important part of the adsorption methods of hazardous contaminants in the field of water and wastewater treatment. Considering those aspects, the main goal of this review is to evaluate and offer a general overview regarding the recent progress on hydroxyapatite-based adsorbents in this particular area. Developing the current review, an attempt has been made to give appropriate recognition to the most recent data regarding the synthesis methods and targeted pollutants, including important information regarding the synthesis methods and precursors, morphological characteristics of the adsorbent materials and effectiveness of process (Scheme 1 depicts a flow diagram of the structure of this review). As the industry is continuously searching for alternative methods to decrease the environmental burden of both the pollutants present and the methods applied for depollution, this review also attempts to present the use of hydroxyapatite with natural origin, along with materials obtained from synthetic precursors, mechanistic aspects, as well as conclusions regarding the current limitations and future perspectives.

In order to select the works presented in the current review, the popular database Scopus was used, applying as keywords “hydroxyapatite”, “adsorption”, “heavy metals” and as search criteria works published after 2019. The works returned were manually checked in order to remove false-positive results, and a decision regarding their insertion in the review was made after carefully reading of the in extenso paper, considering several factors, including the relevancy for the envisaged research area, characterization of the adsorbent materials and the complexity of the study.

## 2. Adsorption Process Modeling and Mechanism

Evaluation of adsorption processes and adsorbents is based on a thorough modeling using different kinetic models, equilibrium isotherms and thermodynamic data [9]. Together with physicochemical characterization and correlated with operational parameters (temperature, pH, pollutant concentration, adsorbent dose, etc.), these approaches allow a complete image of adsorbent performances and, consequently, process optimization. Several kinetic models [10,11] are primarily used for obtaining relevant insights in adsorption mechanism and rate-controlled steps (mass transport and/or reaction processes, the time to reach equilibrium state, etc.). Generally, for heavy metal adsorption on hydroxyapatite or modified hydroxyapatite, the pseudo-second-order model is more appropriate for lower pollutant concentration while for higher concentration the pseudo-first-order model is in good concordance with experimental data. Adsorption isotherms represent the main approach to assess the optimal adsorption capacity and the interactions between solid and the pollutant from liquid phase and evaluate the distribution between them. There are several mathematical equations [12] applied for fitting the experimental data although the most used are Langmuir and Freundlich in linear or non-linear form, since they satisfactorily cover two general assumptions regarding the sorbate- adsorbent system. For the Langmuir model, the main assumption is that binding sites have similar affinity for heavy metal ions, while the Freundlich model refers to heterogeneous systems where adsorption sites have different affinities for sorbate species. Thermodynamic data are one of the most important methods for characterizing the adsorption process, especially since temperature is an important operational parameter. The Van’t Hoff Equation (1) allows us to determine the thermodynamic functions, such as entropy, enthalpy and free changes in energy. From these data, it can be determined if the process is spontaneous (Δ*H*^0^ < 0, Δ*S*^0^ > 0, Δ*G*^0^ < 0) or non-spontaneous (Δ*H*^0^ > 0, Δ*S*^0^ < 0, Δ*G*^0^ > 0), and, consequently, the performance of the adsorption process can be evaluated.

(1) $$\frac{d(lnK_{eq)}}{dT}=\frac{dH}{RT^{2}}$$

where *K_eq_* = *q_e_*/*C* [11].

Applying different models/equations to experimental data is the first step which is followed by selecting the one that fits the best. This is usually accomplished by comparing the correlation of regression (R^2^) parameter, which is employed to select the appropriate model. However, there are several error functions which better compensate the experimental data error (Table 1) and can be applied to both kinetical or equilibrium data and are critical for process optimization. Rahman et al. [12] observed that adsorption of Pb^2+^, Fe^2+^, and Zn^2+^ on *Kappaphycus* sp. followed a Langmuir model according to the R^2^ parameter while applying another error function, the best fit was for the Temkin model. In addition, the best fit for the Langmuir model was found for Cd(II) adsorption on modified HAP [12] using the sum of square error and hybrid functions.

Several low-cost materials were successfully applied in adsorption studies. Table 2 presents a selection of some materials, as well as their maximum adsorption capacity for comparison purposes.

## 3. Application of Natural-Derived Hydroxyapatite for the Removal of Heavy Elements

Hydroxyapatite is, first of all, a natural occurring mineral. As such, the use of natural-derived hydroxyapatite (HAP) is encountered in several published works. The natural hydroxyapatite can be obtained by the processing of several resources, including animal and fish bones, coral or egg shells [35]. The natural-derived HAP is mainly used in biomedical applications, due to its intrinsic properties, such as the presence of trace elements or its natural structure. Nevertheless, natural HAP can also be encountered in a series of environmental applications, including the ones targeted by the present review, removal of heavy metals (Table 3). In this category, we also mention the studies presenting the separation of one component (typically CaO) from natural sources (wastes of animal origin or algae), followed by other steps to develop the final hydroxyapatite material.

Hassan et al. [36] presented the sorption of Sr(II) on a gamma-radiation composite including natural-derived hydroxyapatite and poly(acrylamide-acrylic acid). The authors proposed a complex mechanism for the Sr(II) uptake, including ion exchange (between the Ca and Sr ions) and surface complexation. The equilibrium data presented suggested that the best fitting isotherm models were Langmuir (for 298 K) and, respectively, Freundlich for 318 and 328 K. In addition, the uptake capacity of the composite was superior to other popular sorbents, including carbon nanotubes, kaolinite, or biosorbents, in the studied concentration range (10–50 mg/L).

HAP sorbent also obtained from bovine bones was applied by Caballero et al. [37] for the removal of Pb(II) in a wide range of concentrations (400–1400 mg/L). Their results suggested that the best fitting isotherm for the adsorption process is represented by the Freundlich isotherm, thus supporting an adsorption in a monolayer. The authors also suggested that the optimum adsorbent concentration was 0.7 g/L, for this concentration being reached a removal efficiency of approx. 100%. Adsorption of Pb(II) was also evaluated by Vahdat et al., using both chicken-derived HAP and a magnetic HAP composite, at lower concentrations (1–10 g/L). The obtained results also suggested a pseudo-second-order model kinetic and the Freundlich isotherm being the best model to describe the process. The higher uptake capacity observed can, in our opinion, be assigned on the one hand to the different origin of the HAP, and, on the other hand, to the different lead concentration used.

Removal of Cu(II) was studied by Ngueagni et al. [44], using as sorbent material hydroxyapatite obtained from the core of ox horns. The study involved metal concentration in the range 100–500 mg/L, while the characteristics of the sorbent were determined by the calcination temperature (400–1100 °C), with the Ca/P ratio varying between 1.22 and 1.61, while the specific surface between 130 and 1 m^2^/g. The copper adsorption process for the sample calcinated at 400 °C was best described by the Langmuir isotherm, reaching a maximum adsorption capacity of 99.98 mg/g, at room temperature and a pH of 5, the phenomenon being governed, according to the authors, by a cation exchange process. The adsorption capacity recorded was superior to other sorbents, such as hazelnut activated carbon or bentonite, but inferior to other more complex materials. The same group utilized the material to study the adsorption of lead and cadmium ions, obtaining similar results, the most promising adsorbent being the sample calcinated at 400 °C, in a process best described by the Langmuir isotherm.

The HAP obtained from bovine femur was applied by Ramdani et al. [47] in sorption studies, using as heavy metals Pb(II) and Cd(II). The obtained results were compared with those resulted for the application of commercial HAP, with superior results for both ions. The natural HAP had superior pore size distribution and pore volume, compared with the commercial sample, and similar specific area. The adsorption processes were found to fit the Langmuir isotherm model for the natural HAP and the Freundlich isotherm model for the commercial sample. This could be explained, in our opinion, by the differences in terms of morphological characteristics between the two samples. A similar approach (with similar results) was applied by the same group [48], who compared the efficiency of natural HAP with the one of commercial HAP for the removal of copper and iron(III) ions. The adsorption process was found to be best fitted by the Langmuir isotherm, and the results were superior for the natural HAP in terms of maximum adsorption capacity.

Fish scales were used by Sricharoen et al. [52] to obtain HAP using an ultrasound-assisted method, with application in the uptake of Hg(II). The material obtained had a high uptake capacity for the targeted ion, superior to several types of complex adsorbents, as presented by the authors, a phenomenon assigned by the authors to the ion exchange with the Ca^2+^ in HAP structure, as well to the electrostatic interactions between the positively charged Hg^2+^ and the HAP surface.

An interesting approach was presented by Bi et al. [50]. Using *Chlorella* powder and a microwave-assisted method, the authors obtained hollow microspheres with multicomponent nanocores, in which the dominant phase was HAP, with the presence of whitmoreite, magnetite and chlorapatite. The material was used for the removal of cadmium ions, with a relatively high adsorption capacity. In addition, the magnetite phase present in the composites allowed the magnetic removal of the composite.

A composite based on natural materials (HAP obtained from bovine cortical bone, chitosan obtained from shrimp shells and snail shell powders) was applied by Bambaeero et al. [59] for the removal of copper and zinc ions. The process obeyed a pseudo-second- order model, while the isotherms that best fitted the experimental data were the Langmuir and Temkin isotherms. The authors reached an ion removal of 90% and 60%, respectively, for 3 mg/L initial ions concentration, 0.02 g of adsorbent and a pH of 5.5.

A particular case regarding the natural-derived hydroxyapatite is represented by the use of natural material to obtain the precursors for the hydroxyapatite synthesis. Typically, the material obtained is CaO (noted in Table 3 as *Ca* precursor), which is used to obtain, by the addition of phosphoric acid (or other P-containing precursors), HAP. This approach was used by Xia et al. [39], HAP being applied for the adsorption of Sr(II). According to their results, the process is best fitted by the Liu isotherm model, reaching a maximum adsorption capacity of over 45 mg/g. Núñez et al. [40] applied HAP obtained by a similar recipe for the removal of lead, cadmium and copper ions. The process was better fitted by the Langmuir model, but most importantly, the authors also performed a selectivity study, suggesting a competitive effect between different ions. The lead ions were preferably adsorbed by HAP, most probably due to their high electronegativity and ionic radius closer to Ca(II). Elsanafeny et al. [55] applied a similar method for obtaining the calcium precursor from eggshells and applied the synthesized HAP as such or in the form of polymer modified HAP, in order to propose a method for the treatment of wastewater containing radioactive cobalt and strontium. The obtained results showed superior ion uptake capacity for the HAP. Using egg shells as a source of CaCO_3_, Zeng et al. [43] obtained an anionic/cationic substituted HAP by an ultrasound-assisted procedure. The partial substitution of Ca^2+^ and PO_4_^3−^ with Na^+^, SiO_4_^4−^ and CO_3_^2−^ led to a macroporous structure with a relatively high pore volume compared to the other presented data. The material was applied for the adsorption of lead and cadmium ions, with superior registered maximum adsorption capacity, by comparison with HAP previously presented, and the authors described the uptake process as a mixture of ion exchange, precipitation and electrostatic interactions. The stability of the adsorbent was also established by regeneration studies, the materials being effective after four regeneration cycles.

## 4. Adsorption of Heavy Metals Using Synthesized Hydroxyapatite

The synthesis by various routes of hydroxyapatite can be achieved, including heterogeneous and homogeneous chemical deposition, hydrothermal synthesis, sol-gel methods, and many others [64]. The chemical synthesis method allows the development of materials with tailored morphological properties and composition for envisaged applications [65]. The recent trend in the development of hydroxyapatite-based materials is represented by the development of as-simple-as-possible synthesis methods and with scale-up possibility, in order to achieve their application at industrial scale. Another important aspect in the synthesis of HAP is represented by the necessity of a homogeneous composition of the final material. Generally speaking, in order to achieve this goal, one approach is represented by the used of highly soluble calcium compounds [64]. Another parameter that can be encountered in most of the synthesis methods is represented by the requirement of a high pH value; the replacement of the classical concentrated ammonia with milder alternatives allows the superior control of the process [64]. Other important aspects to be controlled during the synthesis process, especially for environmental applications, are represented by the particle dimensions, specific surface area and porosity, as well as the presence and potential leaching of other elements (in the case of substituted or doped hydroxyapatite). Table 4 presents some of the most recent published works detailing the application of hydroxyapatite for the uptake of heavy metals.

The most encountered synthesis method is represented by co-precipitation. This method can lead to the development of hydroxyapatite that can be used as such or in complex combination with other materials. For example, Ivanets et al. [67] studied the influence of the crystallinity degree and porous structure on the HAP’s adsorption properties of metals from a multi-component solution (Cd^2+^, Co^2+^, Cu^2+^, Fe^3+^, Ni^2+^, Pb^2+^ and Zn^2+^). The variation in crystallinity and porous structure was achieved by the use of Mg^2+^ ions and hydroxyethylenediphosphonic acid (HDEP). The authors obtained an almost complete adsorption of Cd^2+^, Cu^2+^, Fe^3+^, Pb^2+^, Zn^2+^ within 6 h, at the dose of sorbent 5–10 g/L, when using HAP prepared with Mg^2+^ ions. On the other hand, HAP prepared with HDEP showed highest efficiency for the removal of Cd^2+^, Cu^2+^, Fe^3+^, Pb^2+^, Zn^2+^ ions. The proposed order of adsorbent efficiency was Mg^2+^-prepared HAP > HAP > HDEP-prepared HAP, while the most probable adsorption mechanism is represented by ion-exchange and dissolution-precipitation. The relatively high adsorption capacity of nano-sized HAP is expected, considering that, according to the study of Zheng and Zhang [99], commercial HAP was proven a superior adsorbent against copper and zinc ions in static and cylinder dynamic experiments, compared with other often used materials, such as medical stone, nano-carbon, and biochar.

Other encountered methods are represented by the hydrothermal synthesis, sol-gel, microwave synthesis or ultrasonication. All these methods usually lead to the development of rod-shaped particles, well in the nanometric range, with a good absorption capacity towards heavy metals.

Calcium deficiency was also proven to have a direct impact on the metal uptake capacity of HAP. This was demonstrated by Van Dat et al. [69] who obtained calcium-deficient HAP, by selecting different Ca/P ratio, inferior to the stoichiometric one. According to the presented results, the calcium-deficient HAP has superior metal uptake capacity, compared with the non-deficient HAP. The authors also hypothesized that at lower metal concentration (under 0.01 mol/L), the ion-exchange mechanism was dominant, while at higher concentrations, additional precipitation also occurred. As such, the calcium-deficiency strategy can be applied for enhancing the HAP metal uptake capacity and to provide superior adsorbent characteristics.

Another important parameter is represented by the synthesis temperature. The influence of this parameter on the HAP synthesis using a hydrothermal route, and on the U(VI) uptake capacity was evaluated by Zheng et al. [105]. As the synthesis temperature influenced the final morphology of HAP (being obtained nanosheets, nanoribbons and, at a working temperature of 180 °C, blocky structures), other important characteristics were also influenced, including the surface area and the pore volume, in a direct dependence with the temperature. The U(VI) uptake capacity was also influenced, ranging from 336.58–403.91 mg/g. The adsorption process obeyed a pseudo-second-order kinetic model and a Freundlich adsorption isotherm model.

By using modern techniques, such as electrospinning, other HAP morphologies can be obtained (such as hollow fibers), which also proved to have a good efficiency in the heavy metal uptake [79]. The variation of HAP morphology was evaluated by Zou et al. [72], as a result of changes in pH, reaction temperature and reactant ratio. The authors obtained a large variety of morphologies, including fluffy spongy deposits, porous spheres, solid spheres, nanotubes and the dimensions varied between few nanometers to hundreds of nanometers. The strongest effect on the final morphology was assigned to the pH, while the reactant ratio had little effect on the morphology, only affecting the HAP yield. The porous nanosphere morphology was chosen for further experiments, exhibiting a good adsorption capacity for individual ions (Table 4), while for the complex matrix, involving the presence of all ions, HAP exhibited an uptake capacity >99%, for Hg^2+^, Pb^2+^, Cu^2+^, Ni^2+^ and Co^2+^ ions. The kinetics study, performed using Pb(II) as a model pollutant, revealed that the adsorption process followed a pseudo-second-order kinetic model and a Langmuir isotherm model, with a maximum uptake capacity over 250 mg/g.

The presence of organic pollutants (such as oxytetracycline—OTC) has a different effect on the metal uptake on HAP, depending on the studied metal. As such, the adsorption of copper was found to be greatly increased up to 0.25 mmol/L OTC, the lead uptake to increase up to 0.10 mmol/L OTC, followed by a decrease under the level of single metal presence for 0.25 mmol/L OTC, while the cadmium uptake was not influenced by the OTC presence [74]. The recorded results were explained by the authors through the formation of organic-metallic complexes, with different affinity towards HAP, or the blocking of metal sites by OTC (in the case of lead).

Another important practical aspect is related to the recovery of the adsorbent from the solution. This can be easily achieved by the development of composites based on HAP and a magnetic phase. Several authors presented the incorporation of magnetic phases in HAP (i.e., α, γ-Fe_2_O_3_, Fe) and the application of the obtained magnetic composites for the removal of U(VI), Cd(II), or Cu(II) [84,85,86,87,88,89,90,91,92,93,94,95,96,97,98,99,100,101,102,103,104,105]. In all cases, high maximum adsorption capacities were obtained in processes obeying the pseudo-second-order model and Langmuir isotherm models.

Rodrigues et al. [92] synthesized HAP from calcium hydroxide and phosphoric acid and used it to develop adsorbent materials with hydrotalcite (HT) and multi-wall carbon nanotubes (MWCNT) by ultrasonic and hydrothermal treatment. The authors applied the composites HAP/HT and HAP/HT/MWCNT (at two carbon nanotubes concentrations) for the removal of Cr(VI), in fixed bed column experiments. The experimental results proved a very good uptake capacity (between approx. 4 g/g and 5.8 g/g), increasing with the increase in MWCNT content. The kinetic studies evidenced a indicated a feasible, spontaneous, and endothermic physisorption, which could be applied for the removal of Cr(VI) from leather industry wastewater. A multi-component adsorbent was also proposed by Hokkanen et al. [93], using HAP, cellulose and bentonite clay, for the removal of As(III). The process was proven to follow a pseudo-first-order model and Langmuir isotherm model. Best results were obtained in the pH range 4–7, with the equilibrium being reached within 5 min.

Synthesized HAP can be found in more complex adsorbent structures, such as composites with cyclodextrin, activated carbon, carbon nanotubes, hydrotalcite, bentonite, alendronate or even coated on ceramic support as filtration membranes, with significant adsorbent activity against a series of heavy metals (Cd^2+^, Cu^2+^, Cr^6+^, As^3+^, Pb^2+^) [87,88,89,90,91,92,93,94,95,96,97,98,99,100,101,102,103,104,105,106,107,108,109,110,111,112,113,114,115,116,117,118,119,120,121,122,123,124,125,126,127,128,129,130,131,132,133,134,135,136,137], over a wide range of pH values, metal and adsorbent concentrations.

## 5. Mechanistic Aspects and Current Limitations

**Figure 1:**
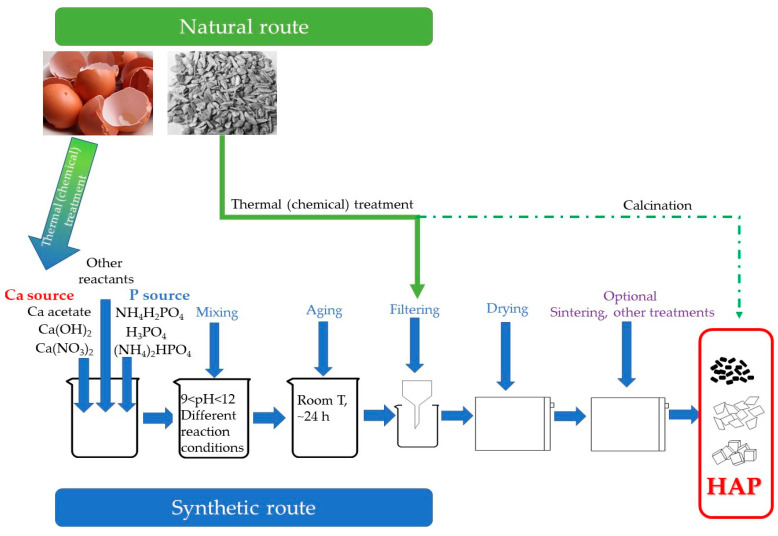
Development of hydroxyapatite-based adsorbents using natural and synthetic route. Schematic presentation.

The development of the proposed adsorbents, either of natural or synthetic origin can be summarized according to Figure 1. The natural route involves two major possible pathways, as previously described.

One is the recovery of the calcium source and its introduction in the synthetic route (as depicted in Figure 1), while another is the direct recovery of hydroxyapatite, trough mechanical, carbonization (and in some cases calcination) of the raw material (typical animal bones). Regarding the synthetic route, a calcium and a phosphorus source are required, followed by different synthesis methods (typically co-precipitation, but other methods, such as hydrothermal, ultrasonication or sol-gel being also encountered), and post-treatments (drying, vacuum-drying, calcination).

**Figure 2:**
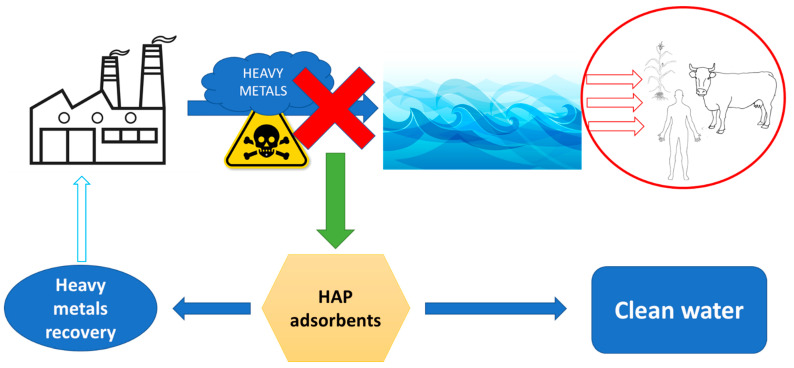
Fate of heavy metals in the environmental and the application of HAP adsorbents for their removal from ecosystems and recovery/reuse.

The presence of heavy metals in water effluents can have a negative influence over very long periods of time [138]. Most of the heavy metals have as provenance various industries, such as textile industry, mining, smelting, fertilizer use, sewage discharge, etc. [139,140]. Regardless their origin, in the absence of appropriate decontamination methods, heavy metal ends up in water effluents, having a direct negative effect on flora, fauna, and finally, human health (Figure 2). Development of appropriate decontamination methods (for example, adsorption) cannot only protect us against these effects, but also allows the recovery of heavy metals, thus lowering the pressure on existent resources.

At the same time, we must consider that the application of efficient decontamination methods has to be affordable, in order to be ready to accept it and implement it for the industry and general public. In this regard, not only the adsorption process is more economically efficient, compared with other methods, but also has smaller associated costs (including operating costs), while the proposed adsorbents are also low-cost, thus offering a viable depollution alternative [141,142,143]. Hydroxyapatite can be easily produced on a large scale, thus allowing the scale-up of the technologies, at the same time being rapidly regenerated, while the metals can be recovered without any expensive procedures.

Hydroxyapatite was also proven to be easily tunable for particular applications, its extraordinary versatility allowing its use in the hardest working conditions. As can be seen from the examples provided above, HAP adsorbents are not only efficient in the up-take of common pollutants (such as Pb^2+^, Cu^2+^, Cd^2+^, Cr^6+^, Ni^2+^, Cd^2+^, Zn^2+^, etc.), but can also uptake metals which are very dangerous and hardly removable by other methods (such as As^3+^) or potential radioactive metals (U^6+^ serving as a model for such elements) in the conditions encountered in practice (in terms of pH value and temperature). Hydroxyapatites-based adsorbents present multifunctional adsorption capacity resulting from FTIR and XRD studies related to modifications observed on hydroxyl, carbonate, phosphate and Ca^2+^ by substitution, ion exchange, complexation or precipitation. Thus, substitution process takes place at Ca^2+^ by metal ions in form of M^2+^, at phosphate groups by different oxyanions AsO_4_^3−^, VO_4_^3−^, etc., and at OH^−^ groups by monovalent anions (F^−^, Cl^−^, etc.). In addition, composite HAP/organic structures can be significantly improved in terms of pollutants species than can be eliminated from aqueous solutions.

Regarding the uptake capacity of the proposed materials, the general conclusion that can be drawn from the presented studies is that the capacity increases with the increase in the specific surface area. Even though the natural hydroxyapatite presents relatively good adsorption capacity towards a variety of metals, its capacity is increased when the approach is used as calcium source, as is the case of lead, for example, recording an increase of Q_max_ from around 250 mg Pb(II)/g for HAP originating from bovine horns to approx. 700 mg/g, for HAP obtained using eggshells as calcium precursor [43,45]. A very good adsorption is achieved using HAP obtained from fish scales for Hg removal (over 200 mg/g) [52]. The adsorption capacity of synthesized HAP is increased not only by comparison with the natural materials, but also with the functionalization of the material or with the incorporation in composites with increased adsorption capacity. For example, the adsorption capacity for esterified HAP reaches approx. 2400 mg Pb(II)/g [81], and is increased up to over 2000 mg U(VI)/g, for the mesoporous HAP, obtained by freeze-drying [136].

Most of the studies concerning the adsorption of the heavy metals on the natural-derived or synthetic HAP suggest that the adsorption process obeys a pseudo-second-order kinetic model, supporting as a main process the chemisorption (with some of the authors also suggesting the presence of the physisorption process, demonstrated by the application of a pseudo-first-order kinetic model), while the most appropriate isotherm to fit the experimental data was the Langmuir isotherm, suggesting a monolayer adsorption (at a fixed number of well-defined sites). The less encountered Freundlich isotherm suggests a different model, presuming that the concentration of the adsorbate on the adsorbent surface increases with its concentration. The Liu isotherm (one of the rarely applied models) is in turn a combination of those two, suggesting that the adsorbate has preferred sites for occupation, but this in turn can be saturated. In our opinion, although less encountered in the literature, this isotherm model should be further studied for the adsorption of heavy metals on HAP adsorbents.

**Figure 3:**
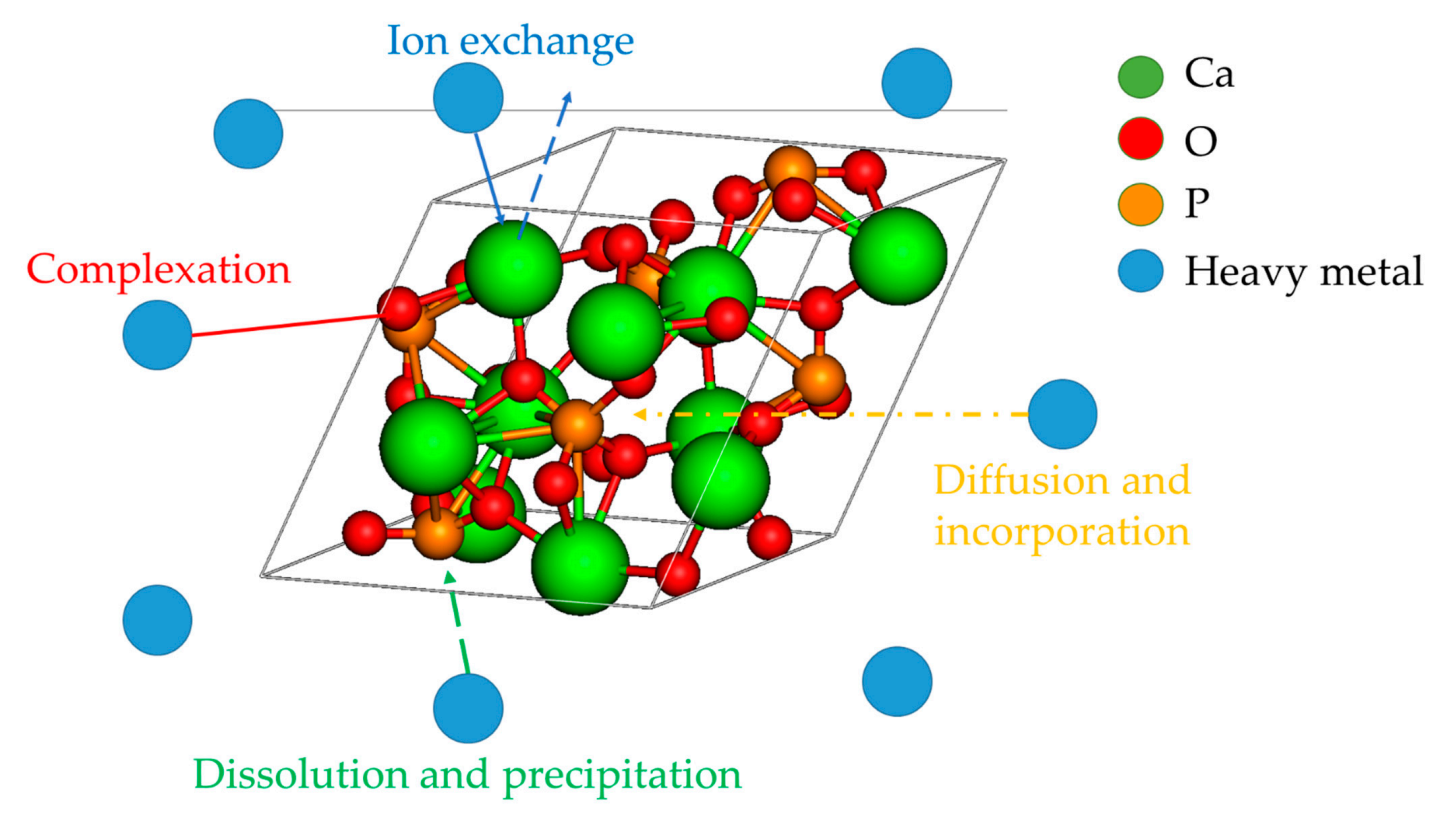
Possible mechanisms of heavy metal uptake by HAP adsorbents, according to the presented literature data.

The widely acceptance of the pseudo-second-order kinetic model for the heavy metal uptake by HAP implies that the sorption process is controlled by chemical reactions, such as ion exchange, surface complexation and/or precipitation, and to a lesser extent by the physical absorption (as would be suggested by the pseudo-first-order kinetic model) (Figure 3).

An important parameter which governs the adsorption mechanism to a large extent is the pH value correlated with pH_PZC_ which predicts to some extent the solid surface charge and metal speciation. Hence, for some pH intervals, in which the solid particle and the pollutant have opposite charges, the electrostatic attraction occurs, while in other conditions, ionic exchange or precipitation are the main adsorption route.

Regeneration of such adsorbents can be easily achieved using, for example, HNO_3_ (0.1 M), Ca(NO_3_)_2_ (0.5 M, pH = 3), or HCl (1.5 M) solutions at room temperature, as demonstrated by Zeng et al. [43], Ma et al. [119], Shen et al. [125] and Ahmed et al. [127], the adsorbent materials preserving their properties after several cycles. Thus, the regeneration step can be achieved in industrial installation, without the need for very complicated equipment. Once recovered, the heavy metals can be reintroduced in the industrial processes. The application of hydroxyapatite-based adsorbent raises another issue that should be addressed more thoroughly in future studies, namely the presence of competition between metallic species for the binding sites, in the case of real-life multicomponent cases, as observed by some of the cited authors [40,41,42,43,44,45,46,47,48,49,50,51,52,53,54,55,56,57,58,59,60,61,62,63,64,65,66,67,68,69,70,71,72,73,74,75,76,77,78,79,80,81,82,83,84,85,86,87,88,89,90,91,92,93,94,95,96,97,98,99,100,101,102,103,104,105,106,107,108,109,110,111,112,113,114,115,116,117,118,119,120,121,122,123] and also observed in studies regarding other natural adsorbents [144].

Another important aspect is related to data presentation in the published studies. In our opinion, future studies regarding the adsorbent properties of HAP should focus on the establishment of the mechanisms through which the processes are taking place, as well as to working in relevant conditions for real systems.

## 6. Conclusions and Future Perspectives

Wastewater treatment remains a critical issue globally, despite various technological advancements and breakthroughs. Apatitic materials are a sustainable, safe and clean method for pollutants’ removal from contaminated environments, with a great advantage over other materials that they can be obtained from natural sources and, more importantly, from waste. Due to their chemical and physical characteristics, they can successfully replace expensive materials, and also can be easily regenerated or have the ability to be converted from used materials into value-added products, and thus apply a zero-waste concept. Preparation methods of apatitic materials is an important step which can direct the efficiency of depollution technology. By referring to the presented data, the most promising synthesis method is represented by the synthetic route, for which the final morphology can be more easily controlled to obtain, for example, mesoporous structures with enhanced adsorption capacity. However, the natural route should not be disregarded, especially when discussing the synthesis process from a bio-economical perspective, considering the re-use of natural wastes. In addition, the particular synthesis method should be selected considering multiple factors, including but not limited to the availability of the raw materials, the targeted metals, and their level on the water matrices. These depollution systems are an important progress for environment treatments providing high efficiency without high costs using easy logistics, with a primordial condition of using optimized tailored materials. Future research is needed in order to obtain optimized materials which can be used in real water systems where the matrix is very complex and, from the point of view of heavy metals, there is an abundance of various competing ions in different concentrations, from traces to enhanced amounts. This challenges, related to the scale-up of technologies from lab scale to commercial adsorption processes, must be addressed through economic limitations and excessive use of chemicals. Even though the process of adsorption is predominant and well-established, the development of low-cost and sustainable materials with enhanced selectivity and stability are still primary challenges. Moreover, detailed studies on the adsorption mechanism are still required for more efficient adsorption process and optimized installations design, and toxicity, selectivity, multi-metal adsorption and reusability are some key challenges to be looked for in future years.

## Figures and Tables

**Table 1:** Several isotherms, kinetic and thermodynamic model equations commonly used in adsorption studies ^1^.

Model	Equation	Ref.
Langmuir	$\frac{C_{e}}{q_{e}}=\frac{1}{K_{L}q_{m}}+\frac{C_{e}}{q_{m}}$	[13]
	$R_{L}=\frac{1}{\left(1+K_{L}C_{0}\right)}$	[14]
Freundlich	$\log q_{e}=\log K_{F}+\frac{1}{n}\log C_{e}$	[15]
Temkin	$q_{e}=\left(\frac{R_{T}}{b_{T}}\right)lnA_{T}+\left(\frac{R_{T}}{b_{T}}\right)C_{e}$	[16]
Dubinin–Radushkevich	$\ln\left(q_{e}\right)=\ln\left(q_{m}\right)-B_{D}\varepsilon^{2}$ $\varepsilon =RT\;\ln\left(1+\frac{1}{C_{e}}\right)$	[17]
	$E=\frac{1}{\sqrt{\left(-2B_{D}\right)}}$	[18]
Pseudo-first-order model	$\log\left(q_{e}-q_{t}\right)=\log q_{e}-\frac{k_{1}t}{2.303}$	[19]
Pseudo-second-order model	$\frac{t}{q_{t}}=\frac{1}{k_{2}q_{e}^{2}}+\frac{t}{q_{e}}$ $h=k_{2}q_{e}^{2}$ $t_{1/2}=\frac{1}{k_{2}q_{e}}$	[20]
Elovich	$\frac{dq_{t}}{d_{t}}=\alpha \;\exp\left(-\beta q_{t}\right)$	[16]
	$q_{t}=\frac{1}{\beta }\left(ln\alpha \beta \right)+\frac{1}{\beta }\ln\left(t\right)$	[21]
Intraparticle diffusion	$q_{t}=k_{d}t^{0.5}+C$	[22]
Thermodynamics	$\Delta G^{0}=-RTlnK_{eq}$ $\Delta G^{0}=\Delta H^{0}-T\Delta S^{0}$ $lnK_{eq}=\frac{\Delta S^{0}}{R}-\frac{\Delta H^{0}}{RT}$ $K_{eq}=\frac{q_{e}}{C_{e}}$	[12,23]

^1^ where *C*_0_ (mg/L): adsorbate initial concentration; *C_e_* (mg/L): adsorbate equilibrium concentration; *q_e_* (mg/g): observed biosorption capacity at equilibrium; *q_m_* (mg/g): maximum biosorption capacity; *K_L_* (L/mg): Langmuir constant related to the energy of adsorption; (*R_L_*): a dimensionless constant, known as separation factor; *K_F_* (mg/g)/(mg/L)^1/*n*^: Freundlich isotherm constant related to the sorption capacity; *n*: a constant which gives an idea of the grade of heterogeneity; *R* (8.314 J/mol): universal gas constant; *T* (°K): absolute temperature; *A_T_* (L/mg): equilibrium binding constant corresponding to the maximum binding energy; *b_T_* (J/mol): Temkin constant related to heat of sorption; *B_D_* (mol^2^/kJ^2^): Dubinin–Radushkevich isotherm constant; *ε*: Polanyi potential related to the equilibrium concentration; *E* (kJ/mol): mean free energy of biosorption; *q_t_* (mg/g): equilibrium adsorption uptake at time, *t*; *k*_1_ (1/min): pseudo-first-order rate constant of adsorption; *k*_2_ (g/mg·min): pseudo-second-order rate constant of adsorption; *h* (mg/g·min): initial adsorption rate; *t*_1/2_ (min): half-adsorption time; *α* (mg/g·min): initial adsorption rate constant; *β* (g/mg): desorption constant; *C* (mg/g): boundary layer diffusion effect; *k_d_* (mg/(g·min^0.5^)): rate constant for intraparticle diffusion; Δ*G*^0^ (kJ/mol): change in Gibbs free energy; Δ*H*^0^ (kJ/mol): change in enthalpy; Δ*S*^0^ (kJ/(mol*K): change in entropy; *K_eq_*: thermodynamic equilibrium constant.

**Table 2:** Maximum adsorption capacity of other low-cost adsorbents.

Adsorbent	Maximum Adsorption Capacity for Heavy Metals (mg Heavy Metal/g)	Ref.
Activated carbon (from coconut)	4.56 mg Pb(II)/g	[24]
Activated carbon (seed hall of palm tree)	3.58 mg Pb(II)/g	[24]
Epichlorohydrin-crosslinked chitosan	34.13 mg Pb(II)/g, 35.46 mg Cu(II)/g, 10.21 mg Zn(II)/g	[25]
Hazelnut husk	13.05 mg Pb(II)/g, 6.645 mg Cu(II)/g	[26]
Muscovite (natural)	0.63 mg Pb(II)/g, 0.618 mg Cu(II)/g, 0.330 mg As(III)/g, 0.791 mg As(V)/g, 0.750 mg Cd(II)/g	[27]
Kaolinite	7.75 mg Pb(II)/g, 4.42 mg Cu(II)/g, 4.95 mg Zn(II)/g	[28]
Modified orange peel	73.53 mg Pb(II)/g, 15.27 mg Cu(II)/g	[29]
Coconut tree sawdust	25 mg Pb(II)/g, 3.89 mg Cu(II)/g, 23.81 mg Zn(II)/g	[30]
Sugarcane bagasse	21.28 mg Pb(II)/g, 3.65 mg Cu(II)/g, 40 mg Zn(II)/g	[30]
Lignin	1865 mg Pb(II)/g, 95 mg Zn(II)/g	[31]
Chitosan	518 mg Cd(II)/g	[32]
Seaweed brown algae	67 mg Cd(II)/g	[33]
*A. nodosum* seaweed	215 mg Cd(II)/g	[33]
Zeolites	155.4 mg Pb(II)/g, 84.3 mg Cd(II)/g, 26 mg Cr(III)/g, 150.4 mg Hg(II)/g	[34]

**Table 3:** Application of natural-derived hydroxyapatite for the removal of heavy elements (references presented in chronological order) ^1^.

Natural Source	Treatment	Application	Heavy Metal	Adsorption Parameters	Ref.
Bovine bones	Boiled, calcinated	Sorption studies using hydroxyapatite/poly (acrylamide-acrylic acid) composite	Sr(II)	Kinetics: pseudo-first-order model; ion exchange predominant model; Q_max_ = 53.59 mg Sr(II)/g	[36]
Bovine bones	Boiled, calcinated	Sorption studies; HAP characteristics: Ca/P ratio = 2, superficial area = 4.106 m^2^/g	Pb(II)	Kinetics: pseudo-second-order model; Q_max_ = 89 mg Pb(II)/g	[37]
Chicken bones	Carbonized, calcinated	Sorption studies using HAP and HAP/Fe_3_O_4_ composites	Pb(II)	Kinetics: pseudo-second-order model; Q_max_ = 105.26 mg Pb(II)/g HAP; Q_max_ = 109.89 Pb(II)/g HAP-Fe_3_O_4_	[38]
Clam shells (Ca precursor)	Dissolved in water and nitric acid, addition of H_3_PO_4_	Sorption studies; HAP: micrometric particle range, SSA 188.5–139.8 m^2^/g	Sr(II)	Kinetics: pseudo-second-order model; Q_max_ = 45.36 mg Sr(II)/g	[39]
Clam shells (Ca precursor)	Grinded, calcinated, addition of H_3_PO_4_	Sorption studies	Pb(II), Cd(II), Cu(II)	Kinetics: pseudo-second-order model; Q_max_ = 265 mg Pb(II)/g; Q_max_ = 64 mg Cd(II)/g, Q_max_ = 55 mg Cu(II)/g	[40]
Mussel shells (Ca precursor)	Grinded, calcinated, addition of NH_4_H_2_PO_4_	Sorption studies	Cd(II)	Kinetics: pseudo-second-order model, Langmuir isotherm; Q_max_ = 62.5 mg Cd(II)/g	[41]
Eggshells (Ca precursor)	Grinded, dissolved in HCl, addition of (NH_4_)_2_HPO_4_	Sorption studies; HAP: hexagonal, 10 nm, SSA: 113 m^2^/g	Pb(II)	Kinetics: pseudo-second-order model; Q_max_ = 129.1 mg Pb(II)/g	[42]
Eggshells (Ca precursor)	Grinded, addition of H_3_PO_4_, Ca(OH)_2_, Na_2_CO_3_ and Na_2_SiO_3_ under ultrasounds to obtain Na-SiCHAP	Sorption studies; hexagonal, 10 nm, SSA: 79.09 m^2^/g, PD 21.32 nm, PV 0.40 cm^3^/g	Pb(II), Cd(II)	Kinetics: pseudo-second-order model, Langmuir isotherm model; Q_max_ = 698.68 mg Pb(II)/g, Q_max_ = 129.60 mg Cd(II)/g	[43]
Bovine horns core	Boiled, acetone soaking, drying, calcination	Sorption studies, using HAP with different characteristics, dependent on the calcination temperature	Cu(II)	Kinetics: pseudo-second-order model; Q_max_ = 99.98 mg Cu(II)/g	[44]
Bovine horns core	Boiled, acetone soaking, drying, calcination	Sorption studies, using HAP with different characteristics, dependent on the calcination temperature	Pb(II), Cd(II)	Kinetics: pseudo-second-order model; Q_max_ = 256.41 mg Pb(II)/g, Q_max_ = 105.26 mg Cd(II)/g	[45]
Fish scales	Soaked in HCl, treated with NaOH, heated	Sorption studies; HAP: Ca/P ratio = 1.96	Pb(II)	100% removal of 0.74 mg/L lead, after 10 min., using 4% HAP	[46]
Bovine femur bone	Washed with water, H_2_O_2_, HNO_3_, bleached, calcinated	Sorption studies, by comparison with commercial HAP; HAP: SSA: 46.8 m^2^/g, PD 25.5 nm, PV 0.18 cm^3^/g	Pb(II), Cd(II)	Kinetics: pseudo-second-order model; Q_max_ = 166.67 mg Pb(II)/g, Q_max_ = 138.89 mg Cd(II)/g	[47]
Bovine femur bone	Washed with water, H_2_O_2_, HNO_3_, bleached, boiled, calcinated	Sorption studies, by comparison with commercial HAP; HAP: SSA: 46.87 m^2^/g, PD 10 nm, PV 0.164 cm^3^/g	Cu(II), Fe(III)	Kinetics: pseudo-second-order model; Q_max_ = 102.35 mg Cu(II)/g, Q_max_ = 87.245 mg Fe(II)/g	[48]
Bovine cow bone	Dried, pyrolyzed, milled	Sorption studies; HAP: SSA: 313.09 m^2^/g, PD 6.46 nm, PV 0.4538 cm^3^/g	Cd(II), Cu(II), Pb(II)	Kinetics: pseudo-second-order model; Langmuir isotherm; Q_max_ = 165.77 mg Cd(II)/g, Q_max_ = 287.58 mg Cu(II)/g, Q_max_ = 558.88 mg Pb(II)/g	[49]
Chlorella powder	Added aq. NaOH and sodium dodecyl sulfate, microwave heated	Sorption studies in the form of hollow microspheres with multicomponent nanocores; HAP: Ca/P ratio = 1.72, PD 32.6 nm	Cd(II)	Kinetics: pseudo-second-order model; Langmuir isotherm; Q_max_ = 116.434 mg Cd(II)/g	[50]
Fish bones	Washed, dried, pulverized, sieved	Sorption studies; particle size 149–325 nm, PD 33–105 nm	Cu(II), Ni(II), Zn(II)	Langmuir isotherm (copper), Freundlich isotherm (nickel and zinc); >95% ion removal (30 mg/kg ion concentration)	[51]
Fish scales	Sonicated, dried, grinded	Sorption studies; HAP: SSA 102.2 m^2^/g, PD 9.14 nm, PV 0.28 cm^3^/g	Hg(II)	Kinetics: pseudo-second-order model; Langmuir isotherm; Q_max_ = 227.27 mg Hg(II)/g	[52]
Eggshells (Ca precursor)	Grinded, calcinated, addition of H_3_PO_4_	Sorption studies using bentonite/CoFe_2_O_4_/HAP composite	Pb(II)	Kinetics: pseudo-second-order model; Langmuir isotherm; Q_max_ = 66 mg Pb(II)/g	[53]
Snail shells (Ca precursor)	Boiled, grinded, calcinated, addition of (NH_4_)_2_HPO_4_	Sorption studies, using HAP and HAP-SiO_2_ composite; HAP: Ca/P ratio = 1.64	Pb(II)	Kinetics: pseudo-second-order model; Langmuir isotherm; Q_max_ = 123 mg Pb(II)/g HAP; Q_max_ = 135.14 mg Pb(II)/g HAP-SiO_2_	[54]
Eggshells (Ca precursor)	Dried, calcinated, addition of H_3_PO_4_	Sorption studies, using HAP and polymeric modified HAP; HAP: Ca/P ratio = 1.63	Co(II), Sr(II)	Kinetics: pseudo-second-order model; Freundlich isotherm; Q_max_ = 43.48 mg Co(II)/g, Q_max_ = 30.4 mg Sr(II)/g	[55]
Chicken thigh bones	Boiled, carbonized, calcinated	Sorption studies using HAP/Fe_3_O_4_/polydopamine composite; HAP: SSA: 16.722 m^2^/g, PV 0.008 cm^3^/g, PD 1.935 nm	Hg(II), Co(II), Ni(II)	Kinetics: Intraparticle diffusion model; Langmuir isotherm; Q_max_ = 51.73 mg Hg(II)/g, Q_max_ = 49.32 mg Co(II)/g, Q_max_ = 48.09 mg Ni(II)/g	[56]
Bovine bones	Boiled, crushed, calcinated	Sorption studies using a dynamic membrane of HAP, *Sargassum glauscens* nanoparticles, chitosan and polyvinyl alcohol	Zn(II), Co(II), Ni(II)	Over 90% removal efficiency	[57]
Fish scales	Boiled in NaOH, dried, calcinated (800 and 900 °C)	Sorption studies; HAP: SSA 88.73/103.46 m^2^/g, PV 0.38/0.36 cm^3^/g, PD 1.64/1.84 nm	Ni(II)	Kinetics: pseudo-first-order model; Langmuir isotherm; Q_max_ = 114.151 mg Ni(II)/g HAP (800 °C), Q_max_ = 181.321 mg Ni(II)/g HAP (800 °C)	[58]
Bovine cortical bones	Carbonized, calcinated	Sorption studies using HAP/chitosan/snail shell powder composite	Cu(II), Zn(II)	Kinetics: pseudo-second-order model; Langmuir/Temkin isotherms; Ion removal: 90%/60% (for 3 mg/L initial ions concentration)	[59]
Camel bones	Dried, grinded, soaked in H_3_PO_4_, treated with HNO_3_ and H_2_O_2_, dried	Sorption studies; HAP consisting material: SSA 19.29 m^2^/g, PV 0.054 cm^3^/g, PD 11.18 nm	V(V)	Kinetics: pseudo-second-order model; Langmuir isotherm; Q_max_ = 19.45 mg V(V)/g	[60]
Chicken bones	Dried, carbonized, calcinated	Sorption studies using HAP, HAP/Fe_3_O_4_ and polydopamine/HAP/Fe_3_O_4,_ composites	Zn(II)	Kinetics: pseudo-second-order model; Freundlich isotherm; Q_max_ = 37.57 mg Zn(II)/g HAP, Q_max_ = 40.07 mg Zn(II)/g Hap-Fe_3_O_4_, Q_max_ = 46.37 mg Zn(II)/g poly-Hap-Fe_3_O_4_	[61]
Eggshells (Ca precursor)	Washed, dried, calcinated, addition of H_3_PO_4_	Sorption studies; HAP: Ca/P ratio = 1.65, SSA 63.7 m^2^/g, PV 0.1512 cm^3^/g	Pb(II)	Kinetics: pseudo-second-order model; Sips isotherm; Q_max_ = 518.46 mg Pb(II)/g	[62]
Eggshells (Ca precursor)	Washed, dried, calcinated, pulverized, addition of HNO_3_ and (NH_4_)_2_HPO_4_	Sorption studies; HAP: Ca/P ratio = 1.74, SSA 32 m^2^/g	Cu(II), Ni(II)	Kinetics: pseudo-second-order model; Freundlich isotherm; Q_max_ = 10.58 mg Cu(II)/g, Q_max_ = 9.53 mg Ni(II)/g	[63]

^1^ where: SSA—specific surface area, PV—pore volume, PD—pore diameter.

**Table 4:** Synthesized hydroxyapatite applied for the removal of heavy metals (references presented in chronological order) ^1^.

Synthesis Method	Precursors, Post-Synthesis Steps	HAP Characteristics	Application	Adsorption Parameters	Ref.
Co-precipitation	Ca(NO_3_)_2_ × 4H_2_O, (NH_4_)_2_HPO_4_, drying at 100 °C for 72 h	Rod shaped, SSA 71.97 m^2^/g	Adsorption of Pb(II), 0–100 mg/L	pH = 5.5, 0.2 g adsorbent, 20 mL solution volume; Langmuir isotherm model, removal efficiency 99.2%	[66]
Co-precipitation	Ca(NO_3_)_2_ × 4H_2_O, H_3_PO_4_, maturation for 24 h	SSA 72–127 m^2^/g, PV 0.233–0.516 cm^3^/g, increasing with the increase of inhibitors concentration.	Adsorption from multicomponent solution—Cd(II), Co(II), Cu(II), Fe(III), Ni(II), Pb(II), Zn(II), 0.13 to 1.28 mmol/g	Pseudo-second-order model, Q_max_ = 25.52 mg Pb(II)/g, Q_max_ = 7.5 mg Cu(II)/g, Q_max_ = 13.26 mg Cd(II)/g, Q_max_ = 0.98 mg Zn(II)/g, Q_max_ = 4.64 mg Ni(II)/g, Q_max_ = 0.59 mg Co(II)/g,	[67]
Dissolution/precipitation	Natural phosphate, HNO_3_, precipitated with NH_4_OH, aged for 24 h, functionalized with sodium benzene-1,3-disulphonate	SSA 100–196.5 m^2^/g, PV 35.3–46.8 cm^3^/g, PD 12–9 nm, in the order of functionalization degree increase	Adsorption of Cd(II), 0–1200 mg/L	pH = 5, pseudo-second-order model, Langmuir isotherm model, Q_max_ = 457.7 mg Cd(II)/g,	[68]
Co-precipitation	Ca(OH)_2_, H_3_PO_4_	Rod-shaped, 20–30 nm diameter, 200–250 nm length	Adsorption of Fe(II), Cu(II), Ni(II), 20–240 mg/L, Cr(VI), 2–30 mg/L	pH = 5.5, Langmuir isotherm model, Q_max_ = 137.23 mg Fe(II)/g, Q_max_ = 128.02 mg Cu(II)/g, Q_max_ = 83.19 mg Ni(II)/g, Q_max_ = 2.92 mg Cr(VI)/g,	[69]
Co-precipitation	Ca(NO_3_)_2_ × 4H_2_O, (NH_4_)_2_HPO_4_	Mesoporous, SSA 49 m^2^/g, PD 14.6 nm	Adsorption of Hg(II), 100–200 mg/L	pH = 3.5–5, Hg(II) up-take: approx. 75%	[70]
Co-precipitation	Ca(NO_3_)_2_ × 4H_2_O, (NH_4_)_2_HPO_4_, thermal treatment	Mesoporous, SSA 100.5 m^2^/g, PD 5.21 nm, PV 0.272 cm^3^/g	Adsorption of Ni(II) and Co(II), 15–1200 mg/L	pH = 4–9, Langmuir isotherm model, Q_max_ = 18.61 mg Ni(II)/g, Q_max_ = 24.27 mg Co(II)/g	[71]
Hydrothermal	Ca(NO_3_)_2_ × 4H_2_O, P_2_O_5_, autoclaved	Various morphologies, dependent on the pH, reaction temperature, and reactant ratio	Adsorption of Pb(II), Cd(II), Cu(II), Co(II), Ni(II), Zn(II), Hg(II)	Efficiency (2 h) 99.61%, 30.95%, 71.08%, 70.36%, 79.56%, 98.60% and 99.90%. Pb(II): pseudo-second-order model, Langmuir isotherm model, Q_max_ = 252.53 mg Pb(II)/g,	[72]
Co-precipitation	Ca(NO_3_)_2_ × 4H_2_O, (NH_4_)_2_HPO_4_, followed by sintering in the presence of polyvinyl alcohol	Granules, SSA 67.6–73 m^2^/g, proportional to the sintering temperature and PVA content	Adsorption of Pb(II), 30–60 mg/L	pH = 2.5–7.5, pseudo-second-order model, Langmuir isotherm model, Q_max_ = 7.99 mg Pb(II)/g	[73]
Co-precipitation	Ca(NO_3_)_2_ × 4H_2_O, (NH_4_)_2_HPO_4_	Rod-shaped, 20–50 nm diameter, 100–200 nm length, SSA 55.24 m^2^/g, PV 0.19 cm^3^/g	Adsorption of Cu(II), Cd(II) and Pb(II), in the presence of oxytetracycline	Langmuir isotherm model, Q_max_ = 55.02 mg Cu(II)/g, Q_max_ = 101.17 mg Cd(II)/g, Q_max_ = 88 mg Pb(II)/g (in the presence of organic substance)	[74]
Sol-gel	Ca(NO_3_)_2_ × 4H_2_O, H_3_PO_4_	SSA 140.41 m^2^/g, PD 10.2 nm	Adsorption of U(VI)	Pseudo-second-order model, Langmuir isotherm model, Q_max_ = 111.4 mg U(VI)/g,	[75]
Co-precipitation	CaCl_2_, Na_2_HPO_4_, in the presence of poly(allylamine hydrochloride)	Flower-like microsphere (diameter of 1–3 μm), nanosheets—8 to 12 nm, SSA 196.4 m^2^/g, PV 0.19 cm^3^/g	Adsorption of Pb(II), Cd(II), and Cu(II)	Pseudo-second-order model, Q_max_ = 210.5 mg Pb(II)/g, Q_max_ = 31.6 mg Cd(II)/g, Q_max_ = 24.9 mg Cu(II)/g	[76]
Co-precipitation	Ca(NO_3_)_2_ × 4H_2_O, H_3_PO_4_, with the post-synthesis addition of hydroxyethylidene diphosphonic acid	SSA 75–207 m^2^/g, PV 0.337–0.779 cm^3^/g, PD 10.9–27.6 nm	Adsorption of Co(II), Sr(II), and Pb(II)	Q_max_ = 38 mg Co(II)/g, Q_max_ = 24.54 mg Sr(II)/g, Q_max_ = 1740.48 mg Pb(II)/g	[77]
Co-precipitation	Ca(NO_3_)_2_ × 4H_2_O, (NH_4_)_2_HPO_4_, with the addition of monododecyl phosphate potassium	Bitter gourd-shaped, 50–120 nm, SSA 77.25 m^2^/g, PV 0.1825 cm^3^/g	Adsorption of Pb(II), Cd(II), and Cr(II)	Q_max_ = 815 mg Pb(II)/g, Q_max_ = 291 mg Cd(II)/g, Q_max_ = 187 mg Cr(II)/g	[78]
Co-precipitation/electrospinning	Ca(NO_3_)_2_ × 4H_2_O, (NH_4_)_2_HPO_4_, capped with polyvinylpyrrolidone, followed by electrospinning and calcination	Nano-fibers, average diameters 500 nm	Adsorption of Cu(II), Cd(II), and Pb(II), 10–300 mg/L	pH = 6, pseudo-second-order model, Langmuir isotherm model, after 2 h, Q_max_ = 23 mg Cu(II)/g, Q_max_ = 36 mg Cd(II)/g, Q_max_ = 93 mg Pb(II)/g	[79]
Co-precipitation	Ca(NO_3_)_2_ × 4H_2_O, (NH_4_)_2_HPO_4_, pH = 10, added 2-bromo- 2-methylpropionic acid, 24 h stirring, acrylonitrile	Not individually determined	Adsorption of Pb(II), 200 mg/L, as n-HAP-g-polyacrylonitrile	pH = 3–7, pseudo-second-order model, Langmuir isotherm model, Q_max_ = 950.5 mg Pb(II)/g	[80]
Co-precipitation	Ca(NO_3_)_2_ × 4H_2_O, (NH_4_)_2_HPO_4_, added acetone and 2-bromo- 2-methylpropionic acid	Esterified HAP: rod-shaped, 10 nm, SSA 66.7 m^2^/g, PV 0.122 cm^3^/g, PD 5.3 nm	Adsorption of Pb(II)	pH = 3–7, pseudo-second-order model, Langmuir isotherm model, Q_max_ = 2398.33 mg Pb(II)/g	[81]
Co-precipitation	CaCO_3_ (limestone), (NH_4_)_2_HPO_4_, post-synthesis doping with Mg^2+^, respectively Sr^2+^	SSA 17.7/71/57 m^2^/g, PV 0.09/0.35/0.28 cm^3^/g, PD 20.35/18.33/19.34 nm	Adsorption of Cu(II)	Pseudo-second-order model, Langmuir isotherm model, Q_max_ = 214.20 mg Cu(II)/g (Mg doped HAP)	[82]
Co-precipitation	Ca(NO_3_)_2_ × 4H_2_O, (NH_4_)_2_HPO_4_, in the presence of sepiolite	Spherical, rod-shaped, 50–100 nm, SSA 55.48–133.54 m^2^/g, PV 0.11–0.48 cm^3^/g, PD 7.94–17.63 nm, varying with the sepiolite amount	Adsorption of Cd(II), 5–50 mg/L, as n-HAP-supported on sepiolite	pH = 3–7, pseudo-second-order model, Sips isotherm model, Q_max_ = 46.81 mg Cd(II)/g	[83]
Co-precipitation	Ca(NO_3_)_2_ × 4H_2_O, H_3_PO_4_, pH = 10, followed by the incorporation of α-Fe_2_O_3_ phase	Rod-shaped, SSA 65 m^2^/g, PV 0.21 cm^3^/g, grain size 10–15 nm	Adsorption of U(VI), 150–300 mg/L	pH = 2–9, pseudo-second-order model, Langmuir isotherm model, Q_max_ = 310 mg U(VI)/g	[84]
Co-precipitation	Ca(NO_3_)_2_ × 4H_2_O, (NH_4_)_2_H PO_4_, in the presence of FeCl_2_ × 4H_2_O	Hollow core mesoporous, shell Fe-HAP, SSA 80.1973 m^2^/g, PV 0.43298 cm^3^/g	Adsorption of Cd(II)	Adsorption up-take 98% (at 296.7 mg/L initial concentration)	[85]
Commercial	Mixing activated carbon, copper nanoparticles, HAP, and sodium alginate	Composites: round structures, SSA 45.1 m^2^/g, PV 0.0443 cm^3^/g	Adsorption of As(III)	Pseudo-second-order model, Langmuir isotherm model, Q_max_ = 39.06 mg As(III)/g	[86]
Co-precipitation	Ca(NO_3_)_2_ × 4H_2_O, (NH_4_)_2_HPO_4_ in the presence of Fe_3_O_4_, followed by β-cyclodextrin binding	Not individually determined	Adsorption of Cd(II) and Cu(II), 20–200 mg/L, as HAP/Fe_3_O_4_ and HAP/Fe_3_O_4_/β-CD	pH = 2–6, pseudo-second-order model, Langmuir isotherm model, Q_max_ = 1000 mg Cd(II)/g composite, Q_max_ = 66.66 mg Cu(II)/g composite	[87]
Co-precipitation	Ca(NO_3_)_2_ × 4H_2_O, (NH_4_)_2_HPO_4_ in the presence of soy bean isolate, calcination	Calcinated at 600 °C: SSA 123.954 m^2^/g	Adsorption of Pb(II), 30–60 mg/L	Pseudo-second-order model, Freundlich isotherm model, Q_max_ = 25.84 mg Pb(II)/g	[88]
Co-precipitation	CaCl_2_, (NH_4_)_2_HPO_4_ in the presence of activated carbon, drying	C-HAP: SSA 60.42 m^2^/g, PV 0.18 cm^3^/g	Adsorption of Pb(II), 0.2–5 mM	pH = 3–7, pseudo-second-order model, Langmuir isotherm model, Q_max_ = 416.67 mg Pb(II)/g	[89]
Co-precipitation	Ca(NO_3_)_2_ × 4H_2_O, Na_2_HPO_4_ in the presence of activated carbon, further coated with turmeric	Nanoflakes	Adsorption of Pb(II), 400–700 mg/kg	pH = 4–7, Langmuir and Freundlich isotherm models gave similar correlations, Q_max_ = 29.4 mg Pb(II)/g	[90]
Commercial	Recrystallization in the presence of pectin	Flower-like particles consisting of sheet-like subunits	Adsorption of Zn(II), 180 mg/L	pH = 5, pseudo-second-order model, Langmuir isotherm model (under 50 mg/L), Freundlich isotherm model (above 60 mg/L), Q_max_ = 330.4 mg Zn(II)/g	[91]
Co-precipitation	Ca(OH)_2_, H_3_PO_4_, addition of hydrotalcite and carbon nanotubes	Not individually determined	Adsorption of Cr(VI), 1040 mg/L	pH = 6, 600 min., pseudo-second-order model, non-linear Freundlich isotherm model	[92]
Co-precipitation	CaCl_2_, NaH_2_PO_4_, in the presence of cellulose and bentonite	Not individually determined	Adsorption of As(III), 0.25–180 mg/L	pH = 2–9.3, pseudo-first-order model, Langmuir isotherm model, Q_max_ = 53.89 mg As(III)/g	[93]
Hydrothermal method	CaCl_2_, sodium dodecyl sulfate, Na_2_CO_3_, in the presence of Fe_3_O_4_ and N-carbon dots	Not individually determined	Adsorption of Pb(II), 10 mg/L	pH = 4–7, pseudo-second-order model, Freundlich adsorption isotherm, Q_max_ = 450.5 mg Pb(II)/g	[94]
Co-precipitation	Ca(OH)_2_, H_3_PO_4_, calcinated, coated over ceramic support	Capsule structure, 100 nm, porous	Adsorption and rejection of Pb(II), 1–10 mg/L	pH = 7.4, Q (g/m^2^) = 0.64 cation rejection 99.6% (at 5 mg/L)	[95]
Co-precipitation	Waste construction putty (as calcium source), (NH_4_)_2_HPO_4_, aging and drying	Leaf-like morphologies, SSA: up to 23.305 m^2^/g, depending on the aging temperature and time	Adsorption of Ni(II)	Q_max_ = 15 mg Ni(II)/g	[96]
Microwave-assisted	CaCl_2_, (NH_4_)_2_HPO_4_, grinded together and microwaved	Rod-shaped, SSA 8.08 m^2^/g, PV 0.05 cm^3^/g, PD 26.82 nm	Adsorption of Pb(II), 5–9 mM	pH = 2–6, pseudo-second-order model, Langmuir adsorption isotherm, Q_max_ = 1438.85 mg Pb(II)/g	[97]
Sol-gel ultrasound assisted	Ca(NO_3_)_2_ × 4H_2_O, (NH_4_)_2_HPO_4_, ultrasonated, aged, freeze-dried	Irregular shapes, SSA 167.93 m^2^/g	Adsorption of Pb(II), 500–2200 mg/L	Pseudo-second-order model, Langmuir adsorption isotherm, Q_max_ = 1303.48 mg Pb(II)/g	[98]
Commercial	-	Rod-shaped	Adsorption of Cu(II), Zn(II)	Freundlich adsorption isotherm model, Q_max_ = 59.03 mg Cu(II)/g, Q_max_ = 55.31 mg Zn(II)/g	[99]
Commercial	-	Micrometric and nanometric HAP	Adsorption of Zn(II), Mn(II), Pb(II), and Cd(II)	Superior effect of nanometric HAP	[100]
Commercial	-	-	Adsorption of Cd(II), in the presence of fulvic acid and montmorillonite colloids	pH = 7, pseudo-second-order model, Freundlich (for pure HAP and in the presence of montmorillonite), Langmuir (in the presence of fulvic acid) adsorption isotherm, Q_max_ = 45–18 mg Cd(II)/g, decreasing with the presence of colloids	[101]
Co-precipitation	Ca(NO_3_)_2_ × 4H_2_O, (NH_4_)_2_HPO_4_, in the presence of citric acid to control morphology, dried	Bundle shaped, length 1 μm, diameter 300 nm	Adsorption of Cd(II), Pb(II)	pH = 4–5, 99.9% adsorption rate	[102]
Co-precipitation	Ca(NO_3_)_2_ × 4H_2_O, (NH_4_)_2_HPO_4_, in the presence of citric acid to control morphology, aged, freeze-dried	Bound and fasciculated nanostructures, length 1 μm, diameter 300 nm, SSA 63.76 m^2^/g	Adsorption of U(VI), 0.02–0.2 g/L	pH = 2–6, pseudo-second-order model	[103]
Co-precipitation	Ca(NO_3_)_2_ × 4H_2_O, (NH_4_)_2_HPO_4_, dried, calcinated (200/800 °C)	HAP (200/800 °C): SSA 58.1/10.8 m^2^/g, PV 0.237/0.014 cm^3^/g, PD 32.7/15.9 nm	Adsorption of Cd(II), 0.02–0.2 g/L	pH = 2–8, pseudo-second-order model, Langmuir adsorption isotherm, Q_max_ = 217.4 mg Cd(II)/g	[104]
Hydrothermal	Ca(NO_3_)_2_ × 4H_2_O, (NH_4_)_2_HPO_4_, different reaction temperatures (120/150/180 °C), autoclaved, dried	Nanosheets, nanoribbons, blocky structures (depending on reaction temperature); SSA 5.31/9.42/10.79 m^2^/g, PV 0.022/0.031/0.033 cm^3^/g, PD 5.861/5.993/5.999 nm	Adsorption of U(VI), 37.36–148.44 mg/mL	pH = 1–7, pseudo-second-order model, Freundlich adsorption isotherm, Q_max_ = 336.58/378.26/403.91 mg U(VI)/g, depending on HAP synthesis temperature	[105]
Co-precipitation	Cu(OH)_2_, Ca(OH)_2_, H_3_PO_4_, dried	Cu-HAP–needle-shaped, SSA 147 m^2^/g	Adsorption of U(VI), 0.5–2.1 μM	55–100% U(VI) up-take	[106]
Co-precipitation	CaCl_2_ × 2H_2_O, (NH_4_)_2_HPO_4_, in the presence of FeCl_2_ × 4H_2_O, aged, dried	Brushite/vivianite mixtures; 80% vivianite: 13.3 m^2^/g	Adsorption of Pb(II), Cr(II), 2 mg/L	pH = 2–8, 98/85% removal at 80% vivianite	[107]
Co-precipitation	CaCl_2_ × 2H_2_O, (NH_4_)_2_HPO_4_, with or without the presence of zoledronate (Z), aged, dried	HAP: rod-shaped, 100 nm, SSA 85.6 m^2^/g, PV 0.41 cm^3^/g, PD 13.79 nm; Z(10%)-HAP: microspherical, 50 nm, SSA 67.46 m^2^/g, PV 0.68 cm^3^/g, PD 33.99 nm	Adsorption of Pb(II), Cu(II), 100–1200/25–200 mg/L	pH = 3–5.5; pseudo-second-order model, Langmuir adsorption isotherm, Q_max_ = 1009.9 mg Pb(II)/g HAP, Q_max_ = 106.96 mg Cu(II)/g HAP; Q_max_ = 1460.14 mg Pb(II)/g Z-HAP, Q_max_ = 226.33 mg Cu(II)/g Z-HAP	[108]
Co-precipitation	Ca(NO_3_)_2_ × 4H_2_O, (NH_4_)_2_HPO_4_, in the presence of attapulgite (A), aged, dried	-	Adsorption of Pb(II), 250–800 mg/L	pH = 2–6; pseudo-second-order model, Langmuir adsorption isotherm, Q_max_ = 421.94 mg Pb(II)/g HAP	[109]
Co-precipitation	Ca(NO_3_)_2_ × 4H_2_O, (NH_4_)_2_HPO_4_, with or without the presence of activated carbon (AC), aged, dried	HAP: needle shaped, SSA 48 m^2^/g; AC-HAP: needle-shapes accumulated over the AC surface, SSA 451 m^2^/g	Adsorption of U(VI), 250–800 mg/L	pH = 7, pseudo-second-order model, Freundlich adsorption isotherm, Q_max_ = 475 mg U(VI)/g HAP, Q_max_ = 464 mg U(VI)/g AC-HAP; Retention of 97.4% U(VI) from tap water at 150 μg/L	[110]
Ultrasonication	CaCO_3_, H_3_PO_4_, mixed post-synthesis with bentonite (B) and ultrasonicated	HAP: semi-spherical, 45 nm, SSA 24.5 m^2^/g, PD 1.1 nm; B-HAP: subspherical hydroxyapatite nanoparticles and bentonite nanosheet, 204.2 m^2^/g, PD 10.2 nm	Adsorption of Pb(II), Ni(II), 5–25 mg/L	pH = 7, pseudo-second-order model, Freundlich adsorption isotherm, Q_max_ = 5.83 mg Pb(II)/g HAP, Q_max_ = 5.99 mg Pb(II)/g B-HAP, Q_max_ = 3.72 mg Ni(II)/g HAP, Q_max_ = 1.01 mg Ni(II)/g B-HAP	[111]
Co-precipitation	ZnFe_2_O_4_ and cetrimonium bromide in ethanol, Ca(NO_3_)_2_ × 4H_2_O added, aged, dried to obtain HAP/ZnFe_2_O_4_	HAP/ZnFe_2_O_4_: rod-shaped crystals of HAP-coated over ZnFe_2_O_4_ nanoparticles, SSA 60.189 m^2^/g	Adsorption of Cd(II), 100–300 mg/L	pH = 3–7, pseudo-second-order model, Freundlich adsorption isotherm, Q_max_ = 114 mg Cd(II)/g	[112]
Co-precipitation	CaCl_2_ × 2H_2_O, (NH_4_)_2_HPO_4_, with or without the presence of alendronate (A), aged, dried	HAP: rod-shaped, 100–200 nm, SSA 80.32 m^2^/g, PV 0.40 cm^3^/g, PD 13.54 nm; A-HAP: globular, 40 nm, SSA 77.87 m^2^/g, PV 0.63 cm^3^/g, PD 29.76 nm	Adsorption of Pb(II), Cu(II), Cd(II), 100–1200/25–200/50–400 mg/L	pH = 5, pseudo-second-order model, Langmuir adsorption isotherm, Q_max_ = 1036.07 mg Pb(II)/g HAP, Q_max_ = 115.74 mg Cu(II)/g HAP, Q_max_ = 234.19 mg Cd(II)/g HAP, Q_max_ = 1529.85 mg Pb(II)/g A-HAP, Q_max_ = 243.31 mg Cu(II)/g A-HAP, Q_max_ = 465.12 mg Cd(II)/g A-HAP	[113]
Sol-gel, electrospinning	Ca(NO_3_)_2_ × 4H_2_O, triethyl phosphite hydrolyzed in ethanol, aged, polyvinylpyrrolidone, followed by electrospinning to obtain HAP/glass fibers (GF), thermal treatment	HAP–GF, segmented fiber structure formed by a chain of crystals, diameter 150 nm, SSA 6.57 m^2^/g, PV 0.025 cm^3^/g, PD 15.75 nm	Adsorption of Cd(II), Pb(II), 60–500 mg/L	pH = 5, pseudo-second-order model, Freundlich adsorption isotherm, Q_max_ = 93.3 mg Cd(II)/g, Q_max_ = 466.98 mg Pb(II)/g	[114]
Commercial	Incorporation of gelatin and zein in HAP by stirring	HAP agglomeration and nodules within the gelatin/zein fibers	Adsorption of Cu(II), 500 mg/L	Q_max_ = 67.8 mg Cu(II)/g, at 50% HAP (increasing with HAP addition)	[115]
Co-precipitation	Calcium and phosphate precursors, cellulose acetate (CA), graphene oxide (GO), magnetic nanoparticles (MNPs), electrospinning to obtain nanofibers	HAP: spherical, 0.9–2.5 μm; CA/HAP: non-oriented nanofibers; MNPs/HAP/GO/CA: random fibers with two diameter groups: 0.6–2.9 μm and 4.3–11.6 μm with spherical grains	Adsorption of Pb(II), Cd(II), 20 mg/L	pH = 8, removal efficiency (MNPs/HAP/GO/CA): 99.1/98.7%	[116]
Co-precipitation	CaCl_2_, Na_3_PO_4_, in the presence of magnetite/bentonite (M-B), N atmosphere, vacuum-dried to obtain M-B-HAP	HAP: flower-like structure; M-B-HAP: rough, compact and undulant surface, SSA 73.72 m^2^/g, PD 9.66 nm, PV 0.026 cm^3^/g	Adsorption of Cd(II), Pb(II), 20–2100 mg/L	pH = 2–10, pseudo-second-order model, Freundlich adsorption isotherm, Q_max_ = 309 mg Cd(II)/g, Q_max_ = 482 mg Pb(II)/g	[117]
Co-precipitation	CaCl_2_ × 2H_2_O, (NH_4_)_2_HPO_4_, cellulose acetate (CA), magnetite nanoparticles (M), graphene oxide (GO) electrospinning	CA-M-GO-HAP: non-oriented fibers, 0.11–0.29 μm, branched from intensive grains (4.57–5.71 μm)	Adsorption of Se(IV), Cr(VI), 25 mg/L	pH = 8, removal efficiency: 96/97.3%	[118]
Hydrothermal	CaCl_2_, (NH_4_)_2_HPO_4_, glucose alkaline solution, redispersed with surfactant, autoclaved, calcination	Mesoporous, 7–15 nm, pores 1.92 nm, SSA 48.81 m^2^/g, PD 1.93 nm, PV 0.38 cm^3^/g	Adsorption of Co(II), Ni(II), Cu(II), Zn(II), Cd(II), 50–1000 mg/L	pH = 5–7, pseudo-second-order model, Freundlich adsorption isotherm, Q_max_ = 299.46 mg Co(II)/g, Q_max_ = 309.35 mg Ni(II)/g, Q_max_ = 248.03 mg Cu(II)/g, Q_max_ = 276.11 mg Zn(II)/g, Q_max_ = 192.93 mg Cd(II)/g	[119]
Co-precipitation	Ca(NO_3_)_2_ × 4H_2_O, (NH_4_)_2_HPO_4_, with Konjac gum (K), xanthan gum (X) or chitosan (C), aged, freeze-dried, calcinated, to obtain HAP aerogels	Uniform porous structure	Adsorption of U(VI), 50–1000 mg/L	Pseudo-second-order model, Langmuir adsorption isotherm, Q_max_ = 2070.3 mg U(VI)/g K-HAP, Q_max_ = 1863.4 mg U(VI)/g X-HAP, Q_max_ = 1446.3 mg U(VI)/g C-HAP	[120]
Co-precipitation	Ca(NO_3_)_2_ × 4H_2_O, (NH_4_)_2_HPO_4_	Cylinder-shaped, 18.29 nm, SSA 75 m^2^/g	Adsorption of Ag(II), 10–100 mg/L	pH = 2–8, pseudo-second-order model, Langmuir adsorption isotherm, Q_max_ = 18.7 mg Ag(II)/g,	[121]
Hydrothermal	CaCl_2_, H_3_PO_4_, with or without the presence of SrCl_2_, autoclaved, to obtain HAP and SrHAP	Nanorods, approx. 4 nm SSA 321.5/378.9 m^2^/g, PD 1.93 nm, PV 0.38 cm^3^/g	Adsorption of Cr(VI), 1000 mg/L	pH = 3.5–7, Langmuir adsorption isotherm, Q_max_ = 322 mg Cr(VI)/g HAP, Q_max_ = 454 mg Cr(VI)/g SrHAP	[122]
Co-precipitation	CaCl_2_, Na_2_HPO_4_, in the presence of oxidized carbon nanotubes (CN)	-	Adsorption of Pb(II), Cd(II), Cu(II), 100 mg/L	pH = 5.5, pseudo-second-order model, Langmuir adsorption isotherm, Q_max_ = 1070–1522 mg Pb(II)/g, Q_max_ = 135–151 mg Cd(II)/g, Q_max_ = 167–180 mg Cu(II)/g (temperature- dependent)	[123]
Co-precipitation	Ca(NO_3_)_2_ × 4H_2_O, (NH_4_)_2_HPO_4_, in the presence of mesoporous carbon (MC), vacuum-aged, thermally treated	HAP: nano-platelets 5/40nm, SSA 101 m^2^/g, PV 0.306 cm^3^/g; MC-HAP: HAP crystallites over amorphous MC, SSA 130 m^2^/g, PV 0.221 cm^3^/g at 8% MC	Adsorption of Cu(II), Ni(II), 15–300 mg/L	pH = 5.5, Elovich adsorption model, Langmuir adsorption isotherm, Q_max_ = 79.5 mg Cu(II)/g, Q_max_ = 18.6 mg Ni(II)/g	[124]
Co-precipitation	Ca(NO_3_)_2_ × 4H_2_O, (NH_4_)_2_HPO_4_, with or without the presence of γ-Fe_2_O_3_ (M), aged, dried	HAP: rod- or needlelike, diameter 5–10 nm, SSA 101 m^2^/g, PV 0.306 cm^3^/g; M-HAP: γ-Fe_2_O_3_ embedded in the needlelike matrix, SSA 130 m^2^/g, PV 0.221 cm^3^/g at 8% MC	Adsorption of Cd(II), 10–100 mg/L	pH = 3–10, pseudo-second-order adsorption model, Langmuir adsorption isotherm, Q_max_ = 277.78 mg Cd(II)/g,	[125]
Co-precipitation	CaCl_2_ × 2H_2_O, (NH_4_)_2_HPO_4_, with or without the presence of amino trimethylposphonate (AT), aged, dried	HAP: SSA 119.75 m^2^/g, PV 0.48 cm^3^/g, PD 15.85; AT-HAP: SSA 92.82 m^2^/g, PV 0.22 cm^3^/g, PD 10.31 nm	Adsorption of Pb(II), Cd(II), 10–1500/50–400 mg/L	pH = 2–6, pseudo-second-order adsorption model, Langmuir adsorption isotherm, Q_max_ = 884.96 mg Pb(II)/g HAP, Q_max_ = 1540.13 mg Pb(II)/g AT-HAP, Q_max_ = 181.82 mg Cd(II)/g HAP, Q_max_ = 367.65mg Cd(II)/g AT-HAP	[126]
Hydrothermal	CaCl_2_ × 2H_2_O, (NH_4_)_2_HPO_4_, in the presence of biochar (BC), autoclaved, dried	HAP on the surface of BC, compact, SSA 157.96 m^2^/g	Adsorption of U(VI), 50 mg/L	pH = 3.5–5.5, pseudo-second-order adsorption model, Langmuir adsorption isotherm, Q_max_ = 428.25 mg U(VI)/g,	[127]
Co-precipitation	Ca(NO_3_)_2_ × 4H_2_O, (NH_4_)_2_HPO_4_, in the presence of polymers: chitosan (CS), carboxymethyl cellulose (CC), alginate (Al), gelatin (Gl), aged, dried	HAP-CS: porous, with rod-shaped particles, 20–50 nm, SSA 191 m^2^/g, PV 0.402 cm^3^/g; HAP-CC: porous, agglomerated spherical particles, SSA 90.6 m^2^/g, PV 0.19 cm^3^/g; HAP-Al: less porous, nanoparticles embedded in polymer, SSA 83.8 m^2^/g, PV 0.12 cm^3^/g; HAP-Gl: less porous, larger particles, SSA 80 m^2^/g, PV 0.33 cm^3^/g;	Adsorption of Pb(II), Cd(II), As(V), 1500/400/1 mg/L	pH = 3–11, pseudo-second-order adsorption model, Freundlich adsorption isotherm, Q_max_ = 514.1 mg Pb(II)/g HAP-CS, Q_max_ = 478.8 mg Pb(II)/g HAP-CC, Q_max_ = 480.3 mg Pb(II)/g HAP-Al, Q_max_ = 579.8 mg Pb(II)/g HAP-Gl, Q_max_ = 114.1 mg Cd(II)/g HAP-CS, Q_max_ = 99 mg Cd(II)/g HAP-CC, Q_max_ = 102.5 mg Cd(II)/g HAP-Al, Q_max_ = 144.9 mg Cd(II)/g HAP-Gl, Q_max_ = 3.38 mg As(VI)/g HAP-CS, Q_max_ = 2.3 mg As(VI)/g JAP-CC, Q_max_ = 2.1 mg As(VI)/g HAP-Al, Q_max_ = 3.17 mg As(VI)/g HAP-Gl	[128]
Co-precipitation	Ca(NO_3_)_2_ × 4H_2_O, (NH_4_)_2_HPO_4_, in the presence of poly-γ-glutamate (PG), aged, dried	PG-HAP: particle dimensions: 79.7–92.8 nm (increased with PG content)	Adsorption of Cu(II), 100 mg/L	pH = 4–6, pseudo-second-order adsorption model, Langmuir adsorption isotherm, Q_max_ = 78.99 mg Cu(II)/g,.for PG/HAP ratio 1/20	[129]
Co-precipitation	(NH_4_)_2_HPO_4_, over solid CaCO_3_, ethanol, aged, dried	Flower-like structures, deposited over calcite, SSA 58.25 m^2^/g, PV 0.0016 cm^3^/g	Adsorption of Zn(II), Cu(II), 5–120 mg/L	pH = 4.6, pseudo-second-order adsorption model, Langmuir adsorption isotherm, Q_max_ = 34.97 mg Zn(II)/g, Q_max_ = 60.24 mg Cu(II)/g,	[130]
Hydrothermal	Ca(OH)_2_, NH_4_H_2_PO_4_, autoclaved, dried, post-synthesis added Fe^0^	Nanorods, diameter 2–10 nm, with Fe^0^ spherical nanoparticles on the surface, SSA 29.03 m^2^/g	Adsorption of Cr(VI), Co(II), Cu(II), Ni(II), 100 mg/L	pH = 5.25, pseudo-first-order adsorption model, Langmuir adsorption isotherm, Removal efficiency: 99.84/99.87/99.33/99.39%	[131]
Not declared	Mixing chitosan (CS), Si slurry and HAP, washed and dried to obtain CS-Si-HAP	Non-homogeneous surface, microstructures, embedded with Si particles, pores in the matrix	Adsorption of Cr(VI), 100 mg/L	pH = 1–7, pseudo-second-order adsorption model, Langmuir adsorption isotherm, Q_max_ = 212.76 mg Cr(VI)/g,	[132]
Co-precipitation	(NH_4_)_2_HPO_4_, over solid limestone, mixed with chitosan (CS), aged, dried	HAP-coated limestone, decreased roughness upon addition of CS; SSA 4.9–11.7 m^2^/g, PV 0.05–0.1 cm^3^/g, PD 25.8–38.2 nm (depending on CS content)	Adsorption of Cu(II), 10–50 mg/L	pH = 3–7, pseudo-second-order adsorption model, Freundlich adsorption isotherm, Q_max_ = 130.75 mg Cu(II)/g, (0.5% CS)	[133]
Co-precipitation	Ca(NO_3_)_2_ × 4H_2_O, KH_2_PO_4_, aged, dried, post-synthesis addition of Fe^0^ and chitin (CH)	Fe-CH-HAP: HAP nanorods, spherical iron particles	Adsorption of Cr(VI), 10–50 mg/L	pH = 3.5–6, pseudo-second-order adsorption model, Langmuir adsorption isotherm, Q_max_ = 39.7 mg Cr(VI)/g,	[134]
Not declared	Mixing Fe_3_O_4_ (M), graphene oxide (GO) and HAP, washed and dried to obtain CS-Si-HAP	Uniform morphology, SSA 158.72 m^2^/g, PV 0.25 cm^3^/g, PD 0.41 nm	Adsorption of Pb(II), 200–800 mg/L	pH = 1–8, pseudo-second-order adsorption model, Langmuir adsorption isotherm, Q_max_ = 249.64 mg Pb(II)/g,	[135]
Freeze-drying-calcination	Ca(NO_3_)_2_ × 4H_2_O, (NH_4_)_2_HPO_4_, in the presence of Konjac gum, lyophilized, calcinated	Porous structure, SSA 118.4 m^2^/g, PV 0.373 cm^3^/g	Adsorption of U(VI), 100 mg/L	pH = 4, pseudo-second-order adsorption model, Langmuir adsorption isotherm, Q_max_ = 2087.6 mg U(VI)/g,	[136]
Co-precipitation	Ca(NO_3_)_2_ × 4H_2_O, (NH_4_)_2_HPO_4_, under ultrasonication, in the presence of biochar (BC), aged, dried	HAP: SSA 157.914 m^2^/g, PV 0.762 cm^3^/g, PD 19.307 nm; BC-HAP: rod-shaped, 50/200 nm, attached on the surface of BC, SSA 144.762 m^2^/g, PV 0.353 cm^3^/g, PD 9.759 nm	Adsorption of Pb(II), Cd(II), 50–2000/50–600 mg/L	pH = 2.05–5.30/2–6.01, pseudo-second-order adsorption model, Langmuir adsorption isotherm, Q_max_ = 1257.03 mg Pb(II)/g HAP, Q_max_ = 770.15 mg Pb(II)/g BC-HAP, Q_max_ = 214.93 mg Cd(II)/g HAP, Q_max_ = 194.61 mg Cd(II)/g BC-HAP	[137]

^1^ where: SS—specific surface area, PV—pore volume, PD—pore diameter.

## Data Availability

Not applicable.

## References

[1] Jaishankar M., Tseten T., Anbalagan N., Mathew B.B., Beeregowda K.N. (2014). Toxicity, mechanism and health effects of some heavy metals. Interdiscip. Toxicol..

[2] Ispas G.C., Manea R., Brazdis R.I., Baroi A.M., Fistos T., Fierascu R.C., Raduly M.F. (2020). Iron Oxide/Phosphatic Materials Composites with Potential Applications in Environmental Protection. Materials.

[3] Jain M., Garg V.K., Kadirvelu K., Sillanpää M. (2015). Adsorption of heavy metals from multi-metal aqueous solution by sunflower plant biomass-based carbons. Int. J. Environ. Sci. Technol..

[4] Osuna-Martínez C.C., Armienta M.A., Tiznado M.E.B., Páez-Osuna F. (2021). Arsenic in waters, soils, sediments, and biota from Mexico: An environmental review. Sci. Total Environ..

[5] Asere T.G., Stevens C.V., Du Laing G. (2019). Use of (modified) natural adsorbents for arsenic remediation: A review. Sci. Total. Environ..

[6] Bano Z., Mazari S., Saeed R.Y., Majeed M.A., Xia M., Memon A.Q., Abro R., Wang F. (2020). Water decontamination by 3D graphene based materials: A review. J. Water Process. Eng..

[7] Sun X., Guo P., Sun Y., Cui Y. (2021). Adsorption of Hexavalent Chromium by Sodium Alginate Fiber Biochar Loaded with Lanthanum. Materials.

[8] Ibrahim M., Labaki M., Giraudon J.-M., Lamonier J.-F. (2020). Hydroxyapatite, a multifunctional material for air, water and soil pollution control: A review. J. Hazard. Mater..

[9] Mongioví C., Morin-Crini N., Lacalamita D., Bradu C., Raschetti M., Placet V., Ribeiro A., Ivanovska A., Kostić M., Crini G. (2021). Biosorbents from Plant Fibers of Hemp and Flax for Metal Removal: Comparison of Their Biosorption Properties. Molecules.

[10] Zhang D., Crini G., Lichtfouse E., Rhimi B., Wang C. (2020). Removal of Mercury Ions from Aqueous Solutions by Crosslinked Chitosan-based Adsorbents: A Mini Review. Chem. Rec..

[11] Gupta A., Sharma V., Sharma K., Kumar V., Choudhary S., Mankotia P., Kumar B., Mishra H., Moulick A., Ekielski A. (2021). A Review of Adsorbents for Heavy Metal Decontamination: Growing Approach to Wastewater Treatment. Materials.

[12] Rahman S., Sathasivam K.V. (2015). Heavy Metal Adsorption ontoKappaphycussp. from Aqueous Solutions: The Use of Error Functions for Validation of Isotherm and Kinetics Models. BioMed Res. Int..

[13] Langmuir I. (1918). The Adsorption of Gases on Plane Surfaces of Glass, Mica AND Platinum. J. Am. Chem. Soc..

[14] Weber T.W., Chakravorti R.K. (1974). Pore and solid diffusion models for fixed-bed adsorbers. AIChE J..

[15] Freundlich H. (1907). Über die Adsorption in Lösungen. Z. Phys. Chem..

[16] Roginsky S.Z., Zeldovich Y.B. (1934). Die Katalische Oxidation von Kohlenmonoxyd Auf Mangandioxyd. Acta Physiochim. URSS.

[17] Dubinin M.M. (1947). The Equation of the Characteristic Curve of Activated Charcoal. Proc. USSR Acad. Sci..

[18] Hobson J.P. (1969). Physical adsorption isotherms extending from ultrahigh vacuum to vapor pressure. J. Phys. Chem..

[19] Lagergren S. (1907). Zur Theorie Der Sogenannten Adsorption Gelöster Stoffe. Z. Chem. Ind. Kolloide.

[20] Ho Y.-S. (1995). Adsorption of Heavy Metals from Waste Streams by Peat. Ph.D. Thesis.

[21] Chien S.H., Clayton W.R. (1980). Application of Elovich Equation to the Kinetics of Phosphate Release and Sorption in Soils. Soil Sci. Soc. Am. J..

[22] Weber J.W., Morris J.C. (1963). Kinetics of Adsorption on Carbon from Solution. J. Sanit. Eng. Div..

[23] Willard Gibbs J. (1873). A method of geometrical representation of the thermodynamic properties of substances by means of surfaces. Transactions of the Connecticut Academy of Arts and Sciences.

[24] Gueu S., Yao B., Adouby K., Ado G. (2007). Kinetics and thermodynamics study of lead adsorption on to activated carbons from coconut and seed hull of the palm tree. Int. J. Environ. Sci. Technol..

[25] Chen A.-H., Liu S.-C., Chen C.-Y. (2008). Comparative adsorption of Cu(II), Zn(II), and Pb(II) ions in aqueous solution on the crosslinked chitosan with epichlorohydrin. J. Hazard. Mater..

[26] Imamoglu M., Tekir O. (2008). Removal of copper (II) and lead (II) ions from aqueous solutions by adsorption on activated carbon from a new precursor hazelnut husks. Desalination.

[27] Yang J.-S., Lee J.Y., Park Y.-T., Baek K., Choi J. (2010). Adsorption of As(III), As(V), Cd(II), Cu(II), and Pb(II) from Aqueous Solutions by Natural Muscovite. Sep. Sci. Technol..

[28] Shahmohammadi-Kalalagh S. (2011). Isotherm and Kinetic Studies on Adsorption of Pb, Zn and Cu by Kaolinite. Casp. J. Environ. Sci..

[29] Lasheen M.R., Ammar N., Ibrahim H.S. (2012). Adsorption/desorption of Cd(II), Cu(II) and Pb(II) using chemically modified orange peel: Equilibrium and kinetic studies. Solid State Sci..

[30] Putra W.P., Kamari A., Yusoff S.N.M., Ishak C.F., Mohamed A., Hashim N., Isa I.M. (2014). Biosorption of Cu(II), Pb(II) and Zn(II) Ions from Aqueous Solutions Using Selected Waste Materials: Adsorption and Characterisation Studies. J. Encapsul. Adsorpt. Sci..

[31] Srivastava S., Singh A., Sharma A. (1994). Studies on the uptake of lead and zinc by lignin obtained from black liquor—A paper industry waste material. Environ. Technol..

[32] Rorrer G.L., Hsien T.Y., Way J.D. (1993). Synthesis of porous-magnetic chitosan beads for removal of cadmium ions from wastewater. Ind. Eng. Chem. Res..

[33] Volesky B., Prasetyo I. (1994). Cadmium removal in a biosorption column. Biotechnol. Bioeng..

[34] Tare V., Chaudhari S., Jawed M. (1992). Comparative Evaluation of Soluble and Insoluble Xanthate Process for Heavy Metal Removal from Wastewaters. Water Sci. Technol..

[35] Akpan E., Dauda M., Kuburi L., Obada D., Dodoo-Arhin D. (2020). A comparative study of the mechanical integrity of natural hydroxyapatite scaffolds prepared from two biogenic sources using a low compaction pressure method. Results Phys..

[36] Hassan H.S., El-Kamash A.M., Ibrahim H.A.-S. (2019). Evaluation of hydroxyapatite/poly(acrylamide-acrylic acid) for sorptive removal of strontium ions from aqueous solution. Environ. Sci. Pollut. Res..

[37] Caballero N., Ozuna P.C., Monteiro M. (2019). Kinetic Analysis of Lead Removal by Natural Hydroxyapatite from Aqueous Solution in High Concentration. Mater. Res..

[38] Vahdat A., Ghasemi B., Yousefpour M. (2019). Synthesis of hydroxyapatite and hydroxyapatite/Fe_3_O_4_ nanocomposite for removal of heavy metals. Environ. Nanotechnol. Monit. Manag..

[39] Xia X., Shen J., Cao F., Wang C., Tang M., Zhang Q., Wei S. (2019). A facile synthesis of hydroxyapatite for effective removal strontium ion. J. Hazard. Mater..

[40] Núñez D., Serrano J.A., Mancisidor A., Elgueta E., Varaprasad K., Oyarzún P., Cáceres R., Ide W., Rivas B.L. (2019). Heavy metal removal from aqueous systems using hydroxyapatite nanocrystals derived from clam shells. RSC Adv..

[41] Meski S., Tazibt N., Khireddine H., Ziani S., Biba W., Yala S., Sidane D., Boudjouan F., Moussaoui N. (2019). Synthesis of hydroxyapatite from mussel shells for effective adsorption of aqueous Cd(II). Water Sci. Technol..

[42] Bernalte E., Kamieniak J., Randviir E.P., Bernalte-García Á., Banks C.E. (2019). The preparation of hydroxyapatite from unrefined calcite residues and its application for lead removal from aqueous solutions. RSC Adv..

[43] Zeng R., Tang W., Ding C., Yang L., Gong D., Kang Z., He Z., Wu Y. (2019). Preparation of anionic-cationic co-substituted hydroxyapatite for heavy metal removal: Performance and mechanisms. J. Solid State Chem..

[44] Ngueagni P.T., Woumfo E.D., Kumar P.S., Siéwé M., Vieillard J., Brun N., Nkuigue P.F. (2020). Adsorption of Cu(II) ions by modified horn core: Effect of temperature on adsorbent preparation and extended application in river water. J. Mol. Liq..

[45] Ngueagni P.T., Kumar P.S., Woumfo E.D., Prasanth S.M. (2020). Adsorption of Pb(II) and Cd(II) ions onto modified biogenic slaughterhouse waste: Equilibrium and kinetic analysis. Int. J. Environ. Anal. Chem..

[46] Omar S., Muhamad M.S., Chuan L.T.E., Rudi N.N., Hamidon N., Hamid N.H.A., Harun H., Sunar N.M., Ali R. (2020). Effect of Hydroxyapatite (HAp) Adsorbent Dosage towards Lead Removal. Int. J. Emerg. Trends Eng. Res..

[47] Ramdani A., Kadeche A., Adjdir M., Taleb Z., Ikhou D., Taleb S., Deratani A. (2020). Lead and cadmium removal by adsorption process using hydroxyapatite porous materials. Water Pract. Technol..

[48] Ramdani A., Taleb Z., Guendouzi A., Kadeche A., Herbache H., Mostefai A., Taleb S., Deratani A. (2020). Mechanism study of metal ion adsorption on porous hydroxyapatite: Experiments and modeling. Can. J. Chem..

[49] Xiao J., Hu R., Chen G. (2020). Micro-nano-engineered nitrogenous bone biochar developed with a ball-milling technique for high-efficiency removal of aquatic Cd(II), Cu(II) and Pb(II). J. Hazard. Mater..

[50] Bi L., Luan X., Geng F., Xu X., Chen Y., Zhang F. (2020). Microwave-assisted synthesis of hollow microspheres with multicomponent nanocores for heavy-metal removal and magnetic sensing. ACS Appl. Mater. Interfaces.

[51] Hernández-Cocoletzi H., Salinas R.A., Águila-Almanza E., Rubio-Rosas E., Chai W.S., Chew K.W., Mariscal-Hernández C., Show P.L. (2020). Natural hydroxyapatite from fishbone waste for the rapid adsorption of heavy metals of aqueous effluent. Environ. Technol. Innov..

[52] Sricharoen P., Limchoowong N., Nuengmatcha P., Chanthai S. (2020). Ultrasonic-assisted recycling of Nile tilapia fish scale biowaste into low-cost nano-hydroxyapatite: Ultrasonic-assisted adsorption for Hg^2+^ removal from aqueous solution followed by “turn-off” fluorescent sensor based on Hg^2+^-graphene quantum dots. Ultrason. Sonochem..

[53] Desalegn Y.M., Andoshe D., Desissa T.D. (2020). Composite of bentonite/CoFe_2_O_4_/hydroxyapatite for adsorption of Pb (II). Mater. Res. Express.

[54] Hariani P.L., Riyanti F., Fatma F., Rachmat A., Herbanu A. (2020). Removal of Pb(II) using Hydroxyapatite from Golden Snail Shell (*Pomacea canaliculata* L.) Modified with Silica. Molekul.

[55] Elsanafeny H.A., Aly M.M.A., Hasan M.A., Lasheen Y.F., Youssef M.A. (2020). Synthesis and polymeric modification of hydroxyapatite from biogenic raw material for adsorptive removal of Co^2+^ and Sr^2+^. J. Radioanal. Nucl. Chem..

[56] Foroutan R., Peighambardoust S.J., Ahmadi A., Akbari A., Farjadfard S., Ramavandi B. (2021). Adsorption mercury, cobalt, and nickel with a reclaimable and magnetic composite of hydroxyapatite/Fe_3_O_4_/polydopamine. J. Environ. Chem. Eng..

[57] Beni A.A., Esmaeili A., Behjat Y. (2021). Invent of a simultaneous adsorption and separation process based on dynamic membrane for treatment Zn(II), Ni(II) and, Co(II) industrial wastewater. Arab. J. Chem..

[58] Ofudje E.A., Adedapo A.E., Oladeji O.B., Sodiya E.F., Ibadin F.H., Zhang D. (2021). Nano-rod hydroxyapatite for the uptake of nickel ions: Effect of sintering behaviour on adsorption parameters. J. Environ. Chem. Eng..

[59] Bambaeero A., Bazargan-Lari R. (2021). Simultaneous removal of copper and zinc ions by low cost natural snail shell/hydroxyapatite/chitosan composite. Chin. J. Chem. Eng..

[60] Ali M.M.S., Imam D.M., El-Nadi Y.A. (2021). Vanadium(V) removal and recovery by adsorption onto modified activated carbon derived from natural hydroxyapatite. J. Iran. Chem. Soc..

[61] Foroutan R., Peighambardoust S.J., Hemmati S., Ahmadi A., Falletta E., Ramavandi B., Bianchi C.L. (2021). Zn^2+^ removal from the aqueous environment using a polydopamine/hydroxyapatite/Fe_3_O_4_ magnetic composite under ultrasonic waves. RSC Adv..

[62] Wei W., Han X., Shao Y., Xie W., Zhang Y., Yao Y., Zhao W., Han R., Li S., Zheng C. (2021). Comparing the effects of humic acid and oxalic acid on Pb(II) immobilization by a green synthesized nanocrystalline hydroxyapatite. Chemosphere.

[63] Ayodele O., Olusegun S.J., Oluwasina O.O., Okoronkwo E.A., Olanipekun E.O., Mohallem N.D., Guimarães W.G., de Gomes B.L.M., de Souza G.O., Duarte H.A. (2021). Experimental and theoretical studies of the adsorption of Cu and Ni ions from wastewater by hydroxyapatite derived from eggshells. Environ. Nanotechnol. Monit. Manag..

[64] Serhiienko A., Dontsova T., Yanushevska O., Lapinskyi A., Krymets G. (2021). Synthesis and characterization of hydroxyapatite and composite based on it with collagen/alginate. Chem. Pap..

[65] Fierascu I., Fierascu R.C., Somoghi R., Ion R.M., Moanţă A., Avramescu S., Damian C.M., Ditu L.M. (2018). Tuned apatitic materials: Synthesis, characterization and potential antimicrobial applications. Appl. Surf. Sci..

[66] Iconaru S.L., Motelica-Heino M., Guegan R., Beuran M., Costescu A., Predoi D. (2018). Adsorption of Pb (II) Ions onto Hydroxyapatite Nanopowders in Aqueous Solutions. Materials.

[67] Ivanets A., Kitikova N., Shashkova I., Roshchina M., Srivastava V., Sillanpää M. (2019). Adsorption performance of hydroxyapatite with different crystalline and porous structure towards metal ions in multicomponent solution. J. Water Process. Eng..

[68] Oulguidoum A., Bouyarmane H., Laghzizil A., Nunzi J.-M., Saoiabi A. (2019). Development of sulfonate-functionalized hydroxyapatite nanoparticles for cadmium removal from aqueous solutions. Colloid Interface Sci. Commun..

[69] Doan V.D., Le V.T., Le H.S., Ta D.H., Nguyen H.T. (2019). Effectiveness of Calcium Deficiency in Nanosized Hydroxyapatite for Removal of Fe(II), Cu(II), Ni(II) and Cr(VI) Ions from Aqueous Solutions. J. Nano Res..

[70] De Resende N.S., Camargo C., Reis P.C., Perez C.A.C., Salim V.M. (2019). Mechanisms of mercury removal from aqueous solution by high-fixation hydroxyapatite sorbents. Int. J. Environ. Sci. Technol..

[71] Ferri M., Campisi S., Gervasini A. (2019). Nickel and cobalt adsorption on hydroxyapatite: A study for the de-metalation of electronic industrial wastewaters. Adsorption.

[72] Zou X., Zhao Y., Zhang Z. (2019). Preparation of hydroxyapatite nanostructures with different morphologies and adsorption behavior on seven heavy metals ions. J. Contam. Hydrol..

[73] Le D.T., Le T.P.T., Do H.T., Vo H.T., Pham N.T., Nguyen T.T., Cao H.T., Nguyen P.T., Dinh T.M.T., Le H.V. (2019). Fabrication of Porous Hydroxyapatite Granules as an Effective Adsorbent for the Removal of Aqueous Pb(II) Ions. J. Chem..

[74] Yuan L., Yan M., Huang Z., He K., Zeng G., Chen A., Hu L., Li H., Peng M., Huang T. (2019). Influences of pH and metal ions on the interactions of oxytetracycline onto nano-hydroxyapatite and their co-adsorption behavior in aqueous solution. J. Colloid Interface Sci..

[75] Su M., Tsang D., Ren X., Shi Q., Tang J., Zhang H., Kong L., Hou L., Song G., Chen D. (2019). Removal of U(VI) from nuclear mining effluent by porous hydroxyapatite: Evaluation on characteristics, mechanisms and performance. Environ. Pollut..

[76] Su Y., Wang J., Li S., Zhu J., Liu W., Zhang Z. (2019). Self-templated microwave-assisted hydrothermal synthesis of two-dimensional holey hydroxyapatite nanosheets for efficient heavy metal removal. Environ. Sci. Pollut. Res..

[77] Shashkova I.L., Ivanets A.I., Kitikova N.V. (2019). Sorption of Co^2+^, Pb^2+^, and Sr^2+^ Ions on Hydroxyapatite, Synthesized in the Presence of Oxyethylidenediphosphonic Acid. Russ. J. Appl. Chem..

[78] Guo H., Jiang C., Xu Z., Luo P., Fu Z., Zhang J. (2019). Synthesis of bitter gourd-shaped nanoscaled hydroxyapatite and its adsorption property for heavy metal ions. Mater. Lett..

[79] Zhou Y., Li S., Wang D., Han X. (2019). Electrospinning Synthesis of Hydroxyapatite Nanofibers Assembled from Nanorods and their Adsorption for Heavy Metal Ions. Pol. J. Environ. Stud..

[80] Wang M., Wang X., Zhang K., Wu M., Wu Q., Liu J., Yang J., Zhang J. (2019). Direct bromination of nano hydroxyapatite strategy towards particle brushes via surface-initiated ATRP for highly efficient heavy metal removal. Polymer.

[81] Wang M., Zhang K., Wu M., Wu Q., Liu J., Yang J., Zhang J. (2019). Unexpectedly High Adsorption Capacity of Esterified Hydroxyapatite for Heavy Metal Removal. Langmuir.

[82] Peng X., Chen W., He Z., Li D., Liu H., Jin H., Zhou G., Xu F. (2019). Removal of Cu(II) from wastewater using doped HAP-coated-limestone. J. Mol. Liq..

[83] Han X., Zhang Y., Li L., Han R., Wang G., Wei W. (2019). Nanosized hydroxyapatite supported on natural sepiolite: A novel adsorbent for Cd(II) removal from simulated groundwater. Mater. Res. Express.

[84] El-Maghrabi H., Younes A.A., Salem A., Rabie K., El-Shereafy E.-S. (2019). Magnetically modified hydroxyapatite nanoparticles for the removal of uranium (VI): Preparation, characterization and adsorption optimization. J. Hazard. Mater..

[85] Guo H., Zhang X., Kang C., Zhang J., Xu Z., Jiang C., Luo P., Fu Z., Ding M., Lv Y. (2019). Synthesis of magnetic Fe-doped hydroxyapatite nanocages with highly efficient and selective adsorption for Cd^2+^. Mater. Lett..

[86] Iqbal J., Shah N.S., Sayed M., Imran M., Muhammad N., Howari F.M., Alkhoori S.A., Khan J.A., Khan Z.U.H., Bhatnagar A. (2019). Synergistic effects of activated carbon and nano-zerovalent copper on the performance of hydroxyapatite-alginate beads for the removal of As3+ from aqueous solution. J. Clean. Prod..

[87] Ansari A., Vahedi S., Tavakoli O., Khoobi M., Faramarzi M.A. (2019). Novel Fe_3_O_4_/hydroxyapatite/β-cyclodextrin nanocomposite adsorbent: Synthesis and application in heavy metal removal from aqueous solution. Appl. Organomet. Chem..

[88] Wang Z., Sun K., He Y., Song P., Zhang D., Wang R. (2019). Preparation of hydroxyapatite-based porous materials for absorption of lead ions. Water Sci. Technol..

[89] Long Y., Jiang J., Hu J., Hu X., Yang Q., Zhou S. (2019). Removal of Pb(II) from aqueous solution by hydroxyapatite/carbon composite: Preparation and adsorption behavior. Colloids Surf. A Physicochem. Eng. Asp..

[90] Jayaweera H.D.A.C., Siriwardane I., De Silva K.M.N., De Silva R.M. (2018). Synthesis of multifunctional activated carbon nanocomposite comprising biocompatible flake nano hydroxyapatite and natural turmeric extract for the removal of bacteria and lead ions from aqueous solution. Chem. Cent. J..

[91] Ni P., Fox J.T. (2019). Synthesis and appraisal of a hydroxyapatite/pectin hybrid material for zinc removal from water. RSC Adv..

[92] Rodrigues E., de Almeida O., Brasil H., Moraes D., dos Reis M. (2019). Adsorption of chromium (VI) on hydrotalcite-hydroxyapatite material doped with carbon nanotubes: Equilibrium, kinetic and thermodynamic study. Appl. Clay Sci..

[93] Hokkanen S., Doshi B., Srivastava V., Puro L., Koivula R. (2019). Arsenic (III) removal from water by hydroxyapatite-bentonite clay-nanocrystalline cellulose. Environ. Prog. Sustain. Energy.

[94] Zhou M., Xie F., Li G., Wang Q., Tang L., Yan M., Bi H., Fei X. (2019). Biocompatible HA@Fe_3_O_4_@N-CDs hybrids for detecting and absorbing lead ion. J. Biomed. Mater. Res. Part A.

[95] Choudhury P.R., Majumdar S., Sarkar S., Kundu B., Sahoo G.C. (2019). Performance investigation of Pb(II) removal by synthesized hydroxyapatite based ceramic ultrafiltration membrane: Bench scale study. Chem. Eng. J..

[96] Thang N.H., Phong D.T. (2020). Characterizations of Hydroxyapatite Synthesized from Calcium Hydroxide and Phosphoric Acid as Adsorbents of Lead in Wastewater. Proceedings of the Materials Science Forum.

[97] Jiang J., Long Y., Hu X., Hu J., Zhu M., Zhou S. (2020). A facile microwave-assisted synthesis of mesoporous hydroxyapatite as an efficient adsorbent for Pb^2+^ adsorption. J. Solid State Chem..

[98] Zhou C., Wang X., Song X., Wang Y., Fang D., Ge S., Zhang R. (2020). Insights into dynamic adsorption of lead by nano-hydroxyapatite prepared with two-stage ultrasound. Chemosphere.

[99] Zheng Y., Zhang J. (2020). Experimental study on the adsorption of dissolved heavy metals by nano-hydroxyapatite. Water Sci. Technol..

[100] Zhang Z., Shi X., Zhang Y., Wang S., Wang M., Wang Y., Weerakoon W.M.S.B., Sanginova O. (2020). Study on immobilization of diatomite, Ca(H_2_PO_4_)_2_, CaCO_3_, HAP and nano-HAP for heavy metal contaminated sediment. Water Qual. Res. J..

[101] Wu M., Mo L., Bi E. (2020). Effects of fulvic acid and montmorillonite colloids at different concentrations on Cd(II) sorption onto nano-hydroxyapatite. Chemosphere.

[102] Tang J., Su M., Wei L., Wei Y., Liang J., Liu Y., Luo Y. (2020). Comprehensive evaluation of the effectiveness on metals recovery and decontamination from MSWI fly ash by an integrating hydrometallurgical process in Guangzhou. Sci. Total Environ..

[103] Shi Q., Su M., Yuvaraja G., Tang J., Kong L., Chen D. (2020). Development of highly efficient bundle-like hydroxyapatite towards abatement of aqueous U(VI) ions: Mechanism and economic assessment. J. Hazard. Mater..

[104] Aouay R., Jebri S., Rebelo A., Ferreira J.M.F., Khattech I. (2020). Enhanced cadmium removal from water by hydroxyapatite subjected to different thermal treatments. J. Water Supply Res. Technol..

[105] Zheng N., Yin L., Su M., Liu Z., Tsang D., Chen D. (2020). Synthesis of shape and structure-dependent hydroxyapatite nanostructures as a superior adsorbent for removal of U(VI). Chem. Eng. J..

[106] Szenknect S., Mesbah A., Descostes M., Maihatchi-Ahamed A., Bonato L., Massonnet M., Ziouane Y., Vors E., Vercouter T., Clavier N. (2020). Uranium removal from mining water using Cu substituted hydroxyapatite. J. Hazard. Mater..

[107] Ahmed M., Mansour S., Ramadan R., Afifi M., Mostafa M.S., El-Dek S., Uskoković V. (2020). Tuning the composition of new brushite/vivianite mixed systems for superior heavy metal removal efficiency from contaminated waters. J. Water Process. Eng..

[108] Fang X., Zhu S., Ma J., Wang F., Xu H., Xia M. (2020). The facile synthesis of zoledronate functionalized hydroxyapatite amorphous hybrid nanobiomaterial and its excellent removal performance on Pb^2+^ and Cu^2+^. J. Hazard. Mater..

[109] Liu G., Liao B., Lu T., Wang H., Xu L., Li Z., Ye C. (2020). Insight into immobilization of Pb^2+^ in aqueous solution and contaminated soil using hydroxyapatite/attapulgite composite. Colloids Surf. A Physicochem. Eng. Asp..

[110] Rout S., Muduli B., Kumar A., Pulhani V. (2020). Removal of Uranium(VI) from Water Using Hydroxyapatite Coated Activated Carbon Powder Nanocomposite. J. Environ. Sci. Health Part A.

[111] El-Nagar D.A., Massoud S.A., Ismail S.H. (2020). Removal of some heavy metals and fungicides from aqueous solutions using nano-hydroxyapatite, nano-bentonite and nanocomposite. Arab. J. Chem..

[112] Das K.C., Dhar S.S. (2020). Removal of cadmium(II) from aqueous solution by hydroxyapatite-encapsulated zinc ferrite (HAP/ZnFe_2_O_4_) nanocomposite: Kinetics and isotherm study. Environ. Sci. Pollut. Res..

[113] Ma J., Xia M., Zhu S., Wang F. (2020). A new alendronate doped HAP nanomaterial for Pb^2+^, Cu^2+^ and Cd^2+^ effect absorption. J. Hazard. Mater..

[114] Roque-Ruiz J.H., Garibay-Alvarado J.A., Medellín-Castillo N.A., Reyes-López S.Y. (2020). Preparation of Electrospun Hydroxyapatite-Glass Fibers for Removal of Cadmium (Cd^+2^) and Lead (Pb^+2^) from Aqueous Media. Water Air Soil Pollut..

[115] Deng L., Li Y., Zhang A., Zhang H. (2020). Nano-hydroxyapatite incorporated gelatin/zein nanofibrous membranes: Fabrication, characterization and copper adsorption. Int. J. Biol. Macromol..

[116] Ahmed M.K., Afifi M., Awwad N.S., Ibrahium H.A. (2020). Pb(II) and Cd(II) removal, mechanical and morphological features of nanofibrous membranes of cellulose acetate containing fillers of hydroxyapatite, graphene oxide, and magnetite. Appl. Phys. A.

[117] UlAinab Q., Zhangab H., Yaseen M., Rasheed U., Liub K., Subhanac S., Tonga Z. (2020). Facile fabrication of hydroxyapatite-magnetite-bentonite composite for efficient adsorption of Pb(II), Cd(II), and crystal violet from aqueous solution. J. Clean. Prod..

[118] Al-Wafi R., Ahmed M., Mansour S. (2020). Tuning the synthetic conditions of graphene oxide/magnetite/hydroxyapatite/cellulose acetate nanofibrous membranes for removing Cr(VI), Se(IV) and methylene blue from aqueous solutions. J. Water Process. Eng..

[119] Ma K., Cui H., Zhou A., Wu H., Dong X., Zu F., Yi J., Wang R., Xu Q. (2021). Mesoporous hydroxyapatite: Synthesis in molecular self-assembly and adsorption properties. Microporous Mesoporous Mater..

[120] Xiong T., Li Q., Liao J., Zhang Y., Zhu W. (2021). Design of hydroxyapatite aerogel with excellent adsorption performance to uranium. J. Environ. Chem. Eng..

[121] Nam P.T., Thanh D.T.M., Phuong N.T., Trang N.T.T., Hong C.T., Anh V.T.K., Lam T.D., Thom N.T. (2021). Adsorption of Ag^+^ Ions Using Hydroxyapatite Powder and Recovery Silver by Electrodeposition. Vietnam J. Chem..

[122] Zhou Y., Li W., Jiang X., Sun Y., Yang H., Liu Q., Cao Y., Zhang Y., Cheng H. (2021). Synthesis of strontium (Sr) doped hydroxyapatite (HAp) nanorods for enhanced adsorption of Cr (VI) ions from wastewater. Ceram. Int..

[123] Li G., Zhang J., Li Y., Liu J., Yan Z. (2021). Adsorption characteristics of Pb(II), Cd(II) and Cu(II) on carbon nanotube-hydroxyapatite. Environ. Technol..

[124] Ferri M., Campisi S., Polito L., Shen J., Gervasini A. (2021). Tuning the sorption ability of hydroxyapatite/carbon composites for the simultaneous remediation of wastewaters containing organic-inorganic pollutants. J. Hazard. Mater..

[125] Shen X., Gao X., Wei W., Zhang Y., Ma L., Liu H., Han R., Lin J. (2021). Combined performance of hydroxyapatite adsorption and magnetic separation processes for Cd(II) removal from aqueous solution. J. Dispers. Sci. Technol..

[126] Zhang Y., Xia M., Wang F., Ma J. (2021). Experimental and theoretical study on the adsorption mechanism of Amino trimethylphosphate (ATMP) functionalized hydroxyapatite on Pb (II) and Cd (II). Colloids Surf. A Physicochem. Eng. Asp..

[127] Ahmed W., Núñez-Delgado A., Mehmood S., Ali S., Qaswar M., Shakoor A., Chen D.-Y. (2021). Highly efficient uranium (VI) capture from aqueous solution by means of a hydroxyapatite-biochar nanocomposite: Adsorption behavior and mechanism. Environ. Res..

[128] Fernando M.S., Wimalasiri A.K.D.V.K., Dziemidowicz K., Williams G.R., Koswattage K.R., Dissanayake D.P., de Silva K.M.N., de Silva R.M. (2021). Biopolymer-Based Nanohydroxyapatite Composites for the Removal of Fluoride, Lead, Cadmium, and Arsenic from Water. ACS Omega.

[129] Chen K.-Y., Zeng W.-Y. (2021). Adsorption of Cu(II) by Poly-γ-glutamate/Apatite Nanoparticles. Polymers.

[130] Gibert O., Valderrama C., Martínez M., Darbra R., Moncunill J., Martí V. (2021). Hydroxyapatite Coatings on Calcite Powder for the Removal of Heavy Metals from Contaminated Water. Water.

[131] Yang W., Xi D., Li C., Yang Z., Lin Z., Si M. (2021). “In-situ synthesized” iron-based bimetal promotes efficient removal of Cr(VI) in by zero-valent iron-loaded hydroxyapatite. J. Hazard. Mater..

[132] Billah R.E.K., Khan M.A., Park Y.-K., Am A., Majdoubi H., Haddaji Y., Jeon B.-H. (2021). A Comparative Study on Hexavalent Chromium Adsorption onto Chitosan and Chitosan-Based Composites. Polymers.

[133] Peng X., Li Y., Liu S., Jiang T., Chen W., Li D., Yuan J., Xu F. (2021). A Study of Adsorption Behaviour of Cu(II) on Hydroxyapatite-Coated-Limestone/Chitosan Composite. J. Polym. Environ..

[134] Rajak J.K., Khandelwal N., Behera M.P., Tiwari E., Singh N., Ganie Z.A., Schäfer T. (2021). Removal of Chromate Ions from Leachate-Contaminated Groundwater Samples of Khan Chandpur, India, Using Chitin Modified Iron-Enriched Hydroxyapatite Nanocomposite. Environ. Sci. Pollut. Res. Int..

[135] Li R., Liu Y., Lan G., Qiu H., Xu B., Xu Q., Sun N., Zhang L. (2021). Pb(II) adsorption characteristics of magnetic GO-hydroxyapatite and the contribution of GO to enhance its acid resistance. J. Environ. Chem. Eng..

[136] Xiong T., Li Q., Liao J., Zhang Y., Zhu W. (2022). Highly enhanced adsorption performance to uranium(VI) by facile synthesized hydroxyapatite aerogel. J. Hazard. Mater..

[137] Zhou C., Song X., Wang Y., Wang H., Ge S. (2022). The sorption and short-term immobilization of lead and cadmium by nano-hydroxyapatite/biochar in aqueous solution and soil. Chemosphere.

[138] Choudhury P.R., Mazumder M.A.J., Al-Attas O., Husain T. (2016). Heavy metals in drinking water: Occurrences, implications, and future needs in developing countries. Sci. Total Environ..

[139] Zhou Q., Yang N., Li Y., Ren B., Ding X., Bian H., Yao X. (2020). Total concentrations and sources of heavy metal pollution in global river and lake water bodies from 1972 to 2017. Glob. Ecol. Conserv..

[140] Fan Y., Chen X., Chen Z., Zhou X., Lu X., Liu J. (2022). Pollution characteristics and source analysis of heavy metals in surface sediments of Luoyuan Bay, Fujian. Environ. Res..

[141] Oulguidoum A., Bouiahya K., Bouyarmane H., Talbaoui A., Nunzi J.-M., Laghzizil A. (2021). Mesoporous nanocrystalline sulfonated hydroxyapatites enhance heavy metal removal and antimicrobial activity. Sep. Purif. Technol..

[142] Gao M., Wang W., Yang H., Ye B.-C. (2019). Hydrothermal synthesis of hierarchical hollow hydroxyapatite microspheres with excellent fluoride adsorption property. Microporous Mesoporous Mater..

[143] Bensalah H., Younssi S.A., Ouammou M., Gurlo A., Bekheet M.F. (2020). Azo dye adsorption on an industrial waste-transformed hydroxyapatite adsorbent: Kinetics, isotherms, mechanism and regeneration studies. J. Environ. Chem. Eng..

[144] LoIacono S., Crini G., Martel B., Chanet G., Cosentino C., Raschetti M., Placet V., Torri G., Morin-Crini N. (2017). Simultaneous removal of Cd, Co, Cu, Mn, Ni, and Zn from synthetic solutions on a hemp-based felt. II. Chemical modification. J. Appl. Polym. Sci..

